# Altered socio-affective communication and amygdala development in mice with protocadherin10-deficient interneurons

**DOI:** 10.1098/rsob.240113

**Published:** 2024-06-19

**Authors:** Tania Aerts, Anneleen Boonen, Lieve Geenen, Anne Stulens, Luca Masin, Anna Pancho, Annick Francis, Elise Pepermans, Geert Baggerman, Frans Van Roy, Markus Wöhr, Eve Seuntjens

**Affiliations:** ^1^ Faculty of Science, Department of Biology, Division of Animal Physiology and Neurobiology, Lab of Developmental Neurobiology, KU Leuven, Leuven 3000, Belgium; ^2^ Faculty of Science, Department of Biology, Division of Animal Physiology and Neurobiology, Lab of Neural Circuit Development and Regeneration, KU Leuven, Leuven 3000, Belgium; ^3^ Developmental Genetics, Department of Biomedicine, University of Basel, Basel 4058, Switzerland; ^4^ Centre for Proteomics, University of Antwerp, Antwerp, Belgium; ^5^ Department of Computer Science, University of Antwerp, Antwerp, Belgium; ^6^ Faculty of Science, Department of Biomedical Molecular Biology; Inflammation Research Center, VIB, Ghent University, Cancer Research Institute Ghent (CRIG) 9000, Belgium; ^7^ Faculty of Psychology and Educational Sciences, Research Unit Brain and Cognition, Laboratory of Biological Psychology, Social and Affective Neuroscience Research Group, KU Leuven, Leuven 3000, Belgium; ^8^ KU Leuven, Leuven Brain Institute, Leuven 3000, Belgium; ^9^ Faculty of Psychology, Experimental and Biological Psychology, Behavioral Neuroscience, Philipps-University of Marburg, Marburg 35032, Germany; ^10^ Center for Mind, Brain and Behavior, Philipps-University of Marburg, Marburg 35032, Germany; ^11^ KU Leuven, Leuven Institute for Single Cell Omics, Leuven 3000, Belgium

**Keywords:** ultrasonic vocalization, non-clustered protocadherin, amygdala development, autism spectrum disorder, proteomics, conditional knockout

## Abstract

Autism spectrum disorder (ASD) is a group of neurodevelopmental conditions associated with deficits in social interaction and communication, together with repetitive behaviours. The cell adhesion molecule protocadherin10 (*PCDH10*) is linked to ASD in humans. *Pcdh10* is expressed in the nervous system during embryonic and early postnatal development and is important for neural circuit formation. In mice, strong expression of *Pcdh10* in the ganglionic eminences and in the basolateral complex (BLC) of the amygdala was observed at mid and late embryonic stages, respectively. Both inhibitory and excitatory neurons expressed *Pcdh10* in the BLC at perinatal stages and vocalization-related genes were enriched in *Pcdh10*-expressing neurons in adult mice. An epitope-tagged *Pcdh10*-HAV5 mouse line revealed endogenous interactions of PCDH10 with synaptic proteins in the young postnatal telencephalon. Nuanced socio-affective communication changes in call emission rates, acoustic features and call subtype clustering were primarily observed in heterozygous pups of a conditional knockout (cKO) with selective deletion of *Pcdh10* in *Gsh2*-lineage interneurons. These changes were less prominent in heterozygous ubiquitous *Pcdh10* KO pups, suggesting that altered anxiety levels associated with *Gsh2*-lineage interneuron functioning might drive the behavioural effects. Together, loss of *Pcdh10* specifically in interneurons contributes to behavioural alterations in socio-affective communication with relevance to ASD.

## 1. Introduction

Autism spectrum disorder (ASD) is a group of deliberating neurodevelopmental conditions that range in both severity and symptoms, which can significantly affect the quality of life. Individuals with ASD are characterized by deficits in two core domains: persistent deficits in social interaction and communication, together with stereotyped, repetitive patterns of behaviour [1]. Comorbid disorders include intellectual disability, epileptic seizures, depression, anxiety, fragile X syndrome and attention deficit hyperactivity disorder [2,3]. Men seem predominantly affected by ASD, showing a fourfold to fivefold increase as compared to women [4,5]. This is partially caused by the underdiagnosis of high-functioning women in research [6].

Rodent models have proven instrumental translational tools to advance our understanding of genetic and environmental factors involved in ASD aetiology. Behavioural phenotyping is a key element of this translational approach and a broad set of sensitive behavioural assays with relevance to the core symptoms and associated features of ASD were developed. These assays target reciprocal social interaction, socio-affective communication, repetitive behaviour, sensory processing, motor and cognitive functions and include the assessment of affective changes, such as anxiety, in juvenile and adult rodents [7–10]. Considering the significance of early developmental profiles in the disease trajectory of neurodevelopmental disorders such as ASD, it is imperative to study behavioural alterations in young offspring. Because the sensory and motor limitations of neonatal pups complicate the assessment of complex behaviours via standard behavioural assays, isolation-induced ultrasonic vocalizations (USV) are commonly used as the primary indicator for socio-affective communicative impairments at early developmental stages, particularly in rodent models for ASD [8,11,12]. Isolation-induced USV are emitted by pups when separated from their mother and littermates, serving important communicative functions to induce maternal retrieval and care [13–15]. The whistle-like pup USV spans a frequency of approximately 30–110 kHz that peaks around postnatal day 6–8 and rapidly decreases thereafter until approximately 2 weeks after birth [16–18]. Anxiolytic drugs, including benzodiazepines and selective serotonin reuptake inhibitors, can decrease the emission rate and duration of these vocalizations, suggesting the mitigation of a presumed anxious state [19–23].

Protocadherin10 (*Pcdh10*) is a cell adhesion molecule of the δ2-subfamily of protocadherins [24]. Protocadherins are believed to mediate intracellular signalling via their unique cytoplasmic tails, in conjunction with their weak adhesive properties [25]. Mutations or copy number variations in *PCDH10* have been linked to ASD [26,27] and comorbid disorders such as Tourette’s syndrome in humans [28]. *Pcdh10* is predominantly expressed in the nervous system, including neurons of the olfactory system, limbic system, visual system, cerebellum and spinal cord [24,29–32]. During early embryonic development, *Pcdh10* expression is most prevalent in the subpallium, increasing in the cortex starting from embryonic day (E) 13.5 [33]. Deletion of *Pcdh10* in mice, by replacing the first exon with a LacZ-neo cassette, resulted in postnatal lethality and severe axonal defects [33]. A recent study using a *Pcdh10* knockout (KO) line without insertion of a *LacZ-neo* cassette refuted these findings [34]. Despite the disparities, *Pcdh10* deficiency was associated with altered behaviours reminiscent of the core symptoms of ASD in both mouse models. This included decreased social approach and alterations in socio-affective communication through USV, together with changes in anxiety levels, impaired fear learning and reduced stress coping [34–36].

The perturbed balance between excitatory neurons and inhibitory interneurons is widely believed to play a prominent role in ASD pathophysiology [37–40]. Interneurons deliver the necessary inhibitory input into local circuits, synchronize projection neuron populations and stabilize network dynamics [41]. Multiple ASD mouse models show a reduced amount or signal intensity of interneurons in the cortex [42–46], hippocampus [43,44], striatum [42,44] and amygdala [47]. One of the hallmarks of ASD mediated by interneurons includes reduced gamma-band oscillations [48]. Heterozygous *Pcdh10* ^+/LacZ^ mice show reduced power of gamma-band activity from the lateral (LA) to basolateral (BLA) amygdala in *ex vivo* brain slices [36] and of gamma-band auditory steady-state responses [49]. The strong expression of *Pcdh10* in interneuron-rich subpallial structures during development and its association with gamma-band oscillations imply that the effects of reduced *Pcdh10* might be caused by an excitation/inhibition imbalance.

In this study, we investigated the role of *Pcdh10* in altered neurodevelopment with relevance to the aetiology of ASD using different mouse models. A *Pcdh10*-HAV5 epitope-tagged mouse line (*Pcdh10* ^tm1(HAV5)Seu^) was created to discover proteins interacting with PCDH10. In addition, we used a first loss-of-function model that has a ubiquitous full-body deletion of *Pcdh10*, while a second model has only a selective deletion of *Pcdh10* in *Gsh2-*lineage interneurons. We found that *Pcdh10* expression in subpallial interneurons increased from embryonic to early postnatal life and was particularly strong in the basolateral amygdala. Conditional KO of *Pcdh10* in *Gsh2-*lineage interneurons reduced this population in the basolateral amygdala. Strikingly, heterozygous loss of *Pcdh10* in the conditional model paralleled and surpassed the behavioural phenotype seen in the ubiquitous KO, marked by an increase in isolation-induced USV emission rates, decreased peak frequency and increased frequency modulation. Additionally, we identified distinct clusters of call subtypes and observed nuanced changes between the genotypes of the conditional *Pcdh10* KO model. Alterations in socio-affective communication in the conditional *Pcdh10* KO suggest *Gsh2*-lineage interneurons drive the observed behavioural effects, and loss of *Pcdh10* specifically in interneurons contributes to behavioural alterations relevant to ASD.

## 2. Methods

### (a) Animals

Conditional *Pcdh10* KO mice were generated from a mutant line containing a floxed first exon of the *Pcdh10* (*Pcdh10* ^tm1.1Fvr^) gene [50], backcrossed in a CD-1/Swiss background for at least nine generations. *Pcdh10* ^fl/+^ mice were crossed with either a RCE line [51] or a *Gsh2*-iCre line [52]. In order to label and selectively delete *Pcdh10* expression in *Gsh2-*interneurons, female *Pcdh10* ^fl/fl^;RCE^fl/fl^ mice were mated with male *Gsh2*-iCre^+/−^;*Pcdh10* ^fl/+^ mice. Homozygous, Cre-positive offspring (*Gsh2*-iCre^+/−^; *Pcdh10* ^fl/fl^; RCE^fl/+^) are referred to as ‘Gsh2:cKO’ mice, while their heterozygous, Cre-positive littermates (*Gsh2*-iCre^+/−^; *Pcdh10* ^fl/+^; RCE^fl/+^) were used as ‘Control’. A breeding scheme is shown in electronic supplementary material, figure S1. To generate ubiquitous *Pcdh10* KO mice, male *Pcdh10* ^fl/+^ mice were crossed with female *Pgk1*-Cre^+/−^ mice [53] and *Pcdh10* ^+/−^ offspring were backcrossed in a CD-1 background for at least three generations. Transgenic *Pcdh10*-HAV5 animals generated via iGONAD were backcrossed for at least three generations before use in experiments to mitigate any potential off-target effects. *Pcdh10*-HAV5 mice were kept in a heterozygous state and mated with wild-type CD-1 mice to maintain the colony. For timed experiments on embryos, mice were mated and females were checked for a copulation plug every morning. This day was considered E0.5. Mice were housed at KU Leuven in a conventional facility with a 14/10 light/dark cycle in a humidity- and temperature-controlled room.

### (b) Genotyping

Polymerase chain reaction (PCR) was performed on purified DNA originating from ear or tail biopsies. Specific amplicons were prepared using several primer pairs (table 1) in combination with GoTaq^©^ DNA Polymerase (Promega).

**Table 1.** Genotyping primers for Pcdh(c)KO mice.

	sequence (5′ → 3′)
*Pcdh10* forward primer	CATGTACCTTCTTCCCACAC
*Pcdh10Flox* reverse primer	GGCATGTGTCAATCAAAGC
*Pcdh10KO* reverse primer	GGTCTGTCAACTAGATAGC
*Cre* forward primer	GAGGGACTACCTCCTCTACC
*Cre* reverse primer	TGCCCAGAGTCATCCTTGGC
*RCE* forward primer 1	CCCAAAGTCGCTCTGAGTTGTTATC
*RCE* forward primer 2	GAAGGAGCGGGAGAAATGGATATG
*RCE* reverse primer	CCAGGCGGGCCATTTACCGTAAG
*Pcdh10HAV5* forward primer	GTCCATCCGTATCCACTTAAAC
*Pcdh10HAV5* forward primer 2	TCCTCGGTCTCGATTCTACGA
*Pcdh10HAV5* reverse primer	CAACACACATCTACTTCCTCCTT

### (c) Tissue processing

Collected postmortem brain samples were isolated in ice-cold phosphate-buffered saline (PBS, 8 mM Na_2_HPO_4_·2H_2_O, 2 mM KH_2_PO_4_, 150 mM NaCl, 3 mM KCl, pH = 7.4), followed by overnight fixation in 4% paraformaldehyde (PFA) in PBS at 4°C. Juvenile pups used for postmortem brain isolation were anaesthetized with an intraperitoneal injection of 50 µl dolethal (sodiumpentobarbital, 2 mg kg^−1^), followed by intracardiac perfusion with saline and 4% PFA. After overnight fixation, samples were washed with PBS and stored in a storage buffer (PBS with 0.4% NaN_3_) at 4°C until use. Brains or tissue used for cell culture or flow cytometry were collected in ice-cold L15^++^ media (Leibovitz’s L-15 media (Gibco) supplemented with 35 mM d-glucose (Sigma) and 2.5 mM HEPES (Gibco)). For protein isolation, brains were collected in ice-cold PBS supplemented with complete, EDTA-free protease inhibitor cocktail tablets (Merck) and snap-frozen in liquid nitrogen before storage at −80°C. Ganglionic eminence (GE) tissue used for quantitative PCR (qPCR) analysis was isolated in ice-cold PBS and stored at −20°C until use.

### (d) Generation of tagged Pcdh10HAV5 mice

Separate crRNA and tracrRNA were ordered from IDT. IDT RNAs were diluted in UltraPure nuclease-free water (Invitrogen) to a concentration of 200 µM and stored at −20°C before use. The repair template containing the desired epitope sequence and homology arms was designed around the cut site and ordered from IDT (see table 2). The antisense repair templates were diluted in nuclease-free water to a concentration of 100 µM upon arrival and stored at −20°C.

**Table 2.** Repair template for the generation of Pcdh10HAV5 mice.

	sequence (5′ → 3′)
*sequence encoding left and right homology arms (normal), linkers (underlined), V5 (bold), NaeI restriction site (italic) and HA (bold and underlined) is shown*	TCTTTATTTATTTATTTTTTCCTAGAATAAACATCAGCGA GGAGGAGGAGGAGGAGGA **GGTAAGCCTATCCCTAACC** **CTCTCCTCGGTCTCGATTCTACG** *AGCGCCGCCGGCGGCT* **ACCCCTACGACGTGCCCGACTACGCC**GGAGGAGGAGGA GGAGGAGCAGAGCTCAGCTATCTAGTTGACAGACCTCGC CGAGTTA

CD-1 wild-type females of at least 12 weeks old were mated with CD-1 wild-type males the day before the procedure, and the presence of a copulation plug (CP) was checked the next morning. Upon the presence of a CP, the iGONAD procedure [54,55] was planned at 16.00 the same day. crRNA and tracrRNA were hybridized for 2 min at 94°C, and an injection mixture was prepared containing 30 µM crRNA/tracrRNA, 1 µg µl^−1^ Cas9 protein and 2 µg µl^−1^ ssODN in opti-MEM (Gibco). To anesthesize the mouse, an intraperitoneal injection of medetomidine (1 mg kg^−1^, Orion) and ketamine (75 mg kg^−1^, Eurovet) was performed. Dorsal fur was removed and an ophthalmic ointment (Terramycin, Pfizer) was applied to the eyes. Two small incisions were made at the level of the ovaries, one through the skin and one through the dorsal muscles. The ovaries with attached oviducts were exposed and genome editing mixture injected into the ampulla. Injection capillaries were prepared from Drummond 1-000-0300 glass capillaries (Drummond) and the Narishige PN-31 horizontal puller (Narishige) with the following settings: heater, 80; magnet-sub, 30; magnet-main, 80. The CUY652P2.5X4 electrode (NepaGene) was subsequently prewetted with PBS and placed around the injected oviducts. The oviduct was then electroporated using the NEPA21 electroporator (NepaGene) with three poring pulses of 50 V, a length of 5 ms and intervals of 50 ms with a decay rate of 10% and polarity switching. Next, three transfer pulses with a voltage of 10 V, a length and interval of 50 ms and a decay rate of 40% with polarity switching were applied. The ovaries and oviducts were placed *in situ* and mouse was given post-surgical care. After the identification of a potential founder mice, relevant PCR amplicons were purified using isopropanol/ethanol precipitation and sent to LGC Genomics for sequencing. Acquired results were then aligned to the desired mutated sequence.

### (e) Paraffin embedding

Fixed brain tissue was placed in cassettes, washed overnight with saline, and then subjected to an alcohol dehydration protocol and overnight paraffin embedding protocol with the Excelsior™ AS Tissue Processor and the HistoStar™ Embedding Workstation. Embedded tissue was placed on a wooden holder and inserted into the Microm HM360 rotary microtome. Coronal sections of 6 µm were collected on SuperFrost^®^ Plus slides (Thermo Fisher Scientific) and dried overnight. Sections were kept at room temperature until use.

### (f) Vibratome sectioning

Fixed brains were embedded in 4% ultrapure agarose (Thermo Fisher Scientific) and submerged in cutting buffer (8 mM Na_2_HPO_4_·2H_2_O, 2 mM KH_2_PO_4_, pH = 7.4) in the Micron HV650 (Thermo Fisher Scientific). Coronal sections of 60 µm were cut using a frequency of 70 Hz, an amplitude of 0.7 mm and a speed of 1.4 mm s^−1^. Sections were collected in a storage buffer and stored at 4°C until use. To analyse eGFP signal in Gsh2:cKO and Gsh2:Control pups, vibratome sections were counterstained with 1:1000 DAPI (4′,6-diamidino-2-phenylindole, 1 mg ml^−1^, Sigma) in PBS and incubated for 20 min in the dark, before mounting with Mowiol (Sigma).

For hybridization chain reaction (HCR) on floating vibratome sections, vibratome sections were cut in an RNAse-free environment; storage buffer and cutting buffer were prepared in autoclaved PBS-DEPC (Acros), agarose was prepared in UltraPure DNase/RNase-Free Distilled Water (Invitrogen) and razor blades and cutting bath were cleaned with RNase AWAY^TM^ Surface Decontaminant (Thermo Fisher Scientific). Sections were collected as described and used for HCR procedures within the week.

### (g) *In situ* hybridization

Ten micrograms of a plasmid containing 807 bp of *Pcdh10* exon 1 sequence was linearized with SacII (NEB) in 1 × CutSmart buffer (NEB). *In vitro* transcription was performed by adding linearized plasmid, Sp6 polymerase (Roche), DIG labelling mix (Roche) and RNase inhibitor (Roche) in 1× reaction buffer (Roche) and incubated overnight at 37°C. Generated probes were purified using Micro Bio-SpinTM P-30 Gel Columns with RNase-free Tris Buffer (BioRad) according to the manufacturer’s protocol and stored at −80°C. *In situ* hybridization (ISH) was performed using an automated system (VENTANA Discovery, Roche). Paraffin sections were first processed in a series of deparaffination, post-fixation, pretreatment and proteinase K digestion steps. Two hundred nanograms of antisense *Pcdh10* probe in Ribohybe (Roche) was added to the sections, followed by denaturation (6 min, 90°C) and hybridization (6 h, 70°C). Sections were washed with 0.1× SSC buffer (Roche) three times at 68°C for 12 min each, and post-fixed in RiboFix (Roche). An anti-DIG-alkaline antibody (Roche) was added to the sections, followed by blocking, the addition of the BCIP/NBT substrate (BlueMap1 and BlueMap2) and incubation for 7 h to form a blue precipitate. Sections were washed, counterstained using the Red Counterstain II kit (4 min, Roche), removed from the automated system and rinsed with distilled water. This was followed by progressive ethanol and xylol dehydration and mounting (Eukitt quick-hardening mounting medium, Sigma).

### (h) Hybridization chain reaction

Hybridization chain reaction (HCR) probe pairs targeting the gene of interest were designed using the Jupyter Notebook application in Anaconda with an optimized script as described in Elagoz *et al*. [56]. Probes were ordered as 50 pmol DNA oligopools from integrated DNA technologies (IDT), dissolved in 100 µl nuclease-free water, aliquoted and stored at −20°C until use. The used protocol was based on the HCR v. 3.0 protocol published by Choi *et al*. [57]. Paraffin sections were processed via progressive deparaffinization, rehydration and permeabilization steps (1:3000 Proteinase K (Roche) in PBS-DEPC, 5 min, 37°C). Sections were then washed in autoclaved milliQ, prehybridized by adding probe hybridization buffer to the sections and incubated (30 min, 37°C). 1.2 pmol probe mixture was prepared in probe hybridization buffer per slide, and incubated for at least 16 h at 37°C. Following incubation, excess probe was removed by progressive washing in a series of increasing 5× SSCT buffer (5× SSC, 0.1% Tween20) in probe wash buffer for 15 min each at 37°C. Sections were pre-amplified by adding amplification buffer and incubated (30 min, RT); 9 pmol of the appropriate hairpin 1 and hairpin 2 were separately heated at 95°C for 1.5 min per section, followed by cooling on ice for 5 min and incubation in the dark (30 min, RT) before adding them to the sections. Sections were incubated at room temperature in the dark for at least 16 h. To remove excess hairpins, sections underwent three consecutive washes with 5× SSCT buffer for 10 min each. Sections were counterstained with 1:1000 DAPI in 5× SSCT and incubated for 20 min in the dark. A final wash with 5× SSCT buffer was performed before mounting with Mowiol (Sigma) and storage at 4°C until imaging with Olympus FluoView FV100.

For HCR on vibratome sections, sections were permeabilized with a solution of 0.5% Triton-X100 in autoclaved PBS for 2 h at 37°C in the dark. Sections then underwent prehybridization, probe hybridization, washing, pre-amplification, amplification and DAPI staining with solutions and time schedule as described; however, probe concentration was increased 3.33 times, incubated for at least 20 h and sections were processed as floating sections on a shaking table instead.

### (i) Imaging

All imaging of fluorescent sections was performed using the Olympus FluoView 1000D (confocal) or Leica DM6 (epifluorescence) microscope. Images of ISH were taken with the Leica DM6 in brightfield mode. Images were processed with Fiji/ImageJ [58]. To calculate the percentage of eGFP^+^ interneurons in the BLC, a max projection based on intensity values was made. The BLA/LA outline was traced and a local threshold (Bernsen) was applied to the green channel. The threshold was then converted into a mask and area was measured.

### (j) Flow cytometry and fluorescent-activated cell sorting

Gsh2:Control and Gsh2:cKO embryos were identified by their presence of eGFP signal when observed under a SteREO Discovery.V8 binocular fluorescence microscope (Zeiss). For each experiment, appropriate negative and single-stained controls were taken along. The telencephalon or GEs were isolated, dissociated with forceps and digested at 37°C for 30 min via the addition of digestion solution (6 units of Papain (Sigma) and 9 units of DNase I (Sigma) in L15^++^ media). Cells were washed three times with ice-cold Dulbecco's Phosphate-Buffered Saline (DPBS, Gibco). Thereafter, cells were suspended in 100 µl DPBS and 10 µl 7-AAD (BD Biosciences) was added and incubated for 15 min at room temperature. Then, 400 µl DPBS was added and cells were strained over a Falcon round-bottom tube with a 35 µm Cell Strainer Cap (Corning). Cells were analysed or sorted using the SH800S Cell Sorter equipped with SH800S software (Sony). To analyse flow cytometry data, FlowJo (Becton, Dickinson & Company) or FCS Express 7 (De Novo Software) applications were used. For sorting, a 100 µM sorting chip (Sony) was used and cells were sorted using the semi-purity or purity settings. Approximately 300 000 cells were collected in RNAse-free Microfuge tubes (Thermo Fisher Scientific) containing 500 µl ice-cold DPBS and 1 µl Protector RNAse inhibitor (Roche). For subsequent analysis of mRNA expression via qPCR or bulk RNA sequencing, cells were spun down (1000*g*, 5 min), resuspended in 1 ml TRIzol™ Reagent (Invitrogen), vortexed for 60 s and incubated at room temperature for 5 min. Thereafter, cells were snap-frozen in liquid nitrogen and stored at −80°C. For analysis of DNA content via qPCR, cells were spun down (1000*g*, 5 min), suspended in 200 µl nuclease-free water and frozen at −20°C.

### (k) Quantitative polymerase chain reaction

Total RNA content was extracted from E13.5 GE tissue via the RNeasy™ Plus Mini Kit (Qiagen). Briefly, tissues were thawed, 1 ml TRIzol reagent (Invitrogen) was added and tissue was homogenized using a drill. After a brief incubation with 200 µl chloroform, a density centrifugation at 12 000*g* (3 min) was performed and the upper aqueous phase with RNA was collected and washed with an equal volume of 70% ethanol. RNA was then extracted via the RNeasy MinElute spin column following the manufacturer’s protocol. The RNA content was eluted in 50 µl nuclease-free water and stored at −80°C if not used immediately for cDNA synthesis. For qPCR on sorted cells, cells frozen in Trizol were thawed on ice and RNA was extracted with the RNeasy™ Plus Micro Kit (Qiagen) and eluted in 14 µl nuclease-free water. Separate biological samples with the same genotype were sometimes pooled together to increase RNA yield. For qPCR starting from DNA, DNA was purified via isopropanol/ethanol precipitation and purified DNA was dissolved in 50 µl nuclease-free water.

cDNA was synthesized using the SuperScript^®^ III Reverse transcriptase kit according to the manufacturer’s protocol (Invitrogen). Two units of Ribonuclease H (Invitrogen) were added at the end of the procedure to remove any remaining RNA. A non-template control (NTC) and non-reverse transcriptase control (NRTC) were included in every experiment. qPCR reactions were set up using the SYBR Green supermix containing the SsoAdvanced^TM^ Polymerase (BioRad), 500 nM forward and reverse primer and 1/1–40 diluted cDNA sample. A CFX96 real-time system (BioRad) was used for thermocycling. The used primer pairs are shown in table 3. Primers targeting housekeeping genes *Gapdh* and *Actb* were used for normalization of gene expression. Relative, normalized gene expression was calculated using CFX Maestro software.

**Table 3.** qPCR primers used.

		sequence (5′ → 3′)
*Pcdh10* all isoforms	forward	GTCGTGTACTCGTTCAGTAGTC
	reverse	GATACACTGGGCTCTCTTCATAG
*Pcdh10* long 1 isoform	forward	GATGGCTGTAGAGATGGGTTAG
	reverse	AATAGTTGTACCTTCCGTGTCC
*Pcdh10* long 2 isoform	forward	ACCAAGCCCAAATTATAGACTCA
	reverse	CTCTTTCATTCTGTCGCTCAAAC
*Pcdh10* long 3 isoform	forward	CCCATTTAAGCATTTGTCCATCT
	reverse	ATTCCATATGTAGGCGTTCCTT
*Pcdh10* short isoform	forward	CTCATCTCCTCCATCAGAAAGC
	reverse	CTTCCCTCAGCCACACAAA
*β-actin*	forward	GCTGTATTCCCCTCCATCGTG
	reverse	CACGGTTGGCCTTAGGGTTCA
*Gapdh*	forward	TGTGTCCGTCGTGGATCTGA
	reverse	TTGCTGTTGAAGTCGCAGGAG

### (l) Bulk RNA sequencing

RNA samples of sorted *Gsh2-*lineage cells (eGFP^+^) from control and cKO embryos (E15.5) were submitted for quality control with the BioAnalyzer (Agilent) at the Genomics Core (KU Leuven). Twelve samples were selected for library preparation and sequencing. Libraries were prepared using the QuantSeq 3′ mRNA-Seq Library Prep Kit FWD (Lexogen) and samples were sequenced on the HiSeq4000 (Illumina) in single-read mode at the Genomics Core (KU Leuven). FastQC [59], accessed via the Galaxy webserver (usegalaxy.eu), was used for initial quality check of raw reads. Subsequent data analysis was performed using the Flemish Supercomputer Center. Adapter contamination, polyA and low-quality tails were trimmed using BBDuk [60] in combination with Lexogen i7 and i5 Index Sequences. Indices for genome alignment were generated from the *Mus musculus* GRCm39 genome and GRCm39.108 gene files, available at ensembl.org/Mus_musculus/Info/Index. Reads were aligned with STAR aligner [61] and Featurecounts [62] was used to quantify the reads. Thereafter, differential expression was analysed using DESeq2 [63]. Reads were normalized with size factors calculated by DESeq2.

### (m) Western blotting

Snap-frozen samples were lysed with ASBA lysis buffer [64] containing protease inhibitor cocktail solution (Roche), followed by mechanical homogenization (5 × 10 s), sonification (5 × 10 s), incubation (5 min, 70°C) and centrifugation (15 min, 13 000 r.p.m., 4°C). The supernatant was collected and stored at −80°C. Protein concentration was determined using the Qubit Protein Assay Kit (Invitrogen) and Qubit Instrument (Thermo Fisher Scientific) according to the manufacturer’s protocol.

For western blotting, 20 µg sample was mixed with sample buffer (BioRad) and reducing agent (BioRad). Samples were denatured for 10 min at 70°C and proteins were separated (55 min, 200 V) on a Midi 4–12% Bis-Tris gel (BioRad) in 1× MOPS buffer (BioRad) along with 4 µl of Spectra multicolour Broad Range marker (Thermo Fisher Scientific). Proteins were transferred to a polyvinylidene difluoride membrane (PVDF, BioRad) using the Trans Turbo Blot (BioRad) for 15 min. The PVDF membrane was rehydrated in Tris buffer (5 min, 10 mM Tris, 100 µM thimerosal, 150 mM NaCl, 0,01% Triton-X100, pH = 7.6), and incubated in blocking buffer containing 5% blocking for ECL (GE Healthcare) in Tris buffer for 2 h. Rat anti-PCDH10 antibody (Anti-OL-protocadherin antibody, clone 5G10, Sigma) or mouse anti-GAPDH (Merck) was diluted 1:1000 in blocking buffer, added to the membrane and incubated overnight. Blots were rinsed three consecutive times with Tris buffer (5 min) and 1:10 000 diluted donkey- or goat-anti-rat-horseradish peroxidase (HRP) (Agilent Technologies) or 1:25 000 diluted goat-anti-mouse-HRP (BioRad) in blocking buffer was added (30 min). The blot was subsequently washed with Tris buffer (2 × 5 min) and Tris-stock (1 × 5 min, 50 mM Tris, pH = 7.6) and incubated for 5 min in ECL solution for chemiluminescent detection (Thermo Fisher Scientific). Proteins were visualized using the BioRad Imaging system with Chemi visualization. Protein levels were normalized using total protein stain (TPS) or housekeeping protein GAPDH.

### (n) Co-immunoprecipitation and mass spectrometry

The telencephalon of P7 *Pcdh10*-HAV5 and control pups was collected in ice-cold PBS supplemented with complete, EDTA-free Protease Inhibitor Cocktail Tablets (Merck), cut into small pieces using sterile forceps and submerged in a 0.1% PFA solution for 10 min on ice. Thereafter, tissue was washed three consecutive times with a 1M Tris-solution to quench the crosslinking (pH = 7) and snap-frozen until use. Thawed tissue was lysed in 4 ml lysis buffer (250 mM NaCl, 50 mM tris pH 7.5, 0.25 mM Tris(2-carboxyethyl)phosphine hydrochloride (TCEP), 0.05% *n*-dodecyl-β-ᴅ-maltoside supplemented with complete EDTA-free Protease Inhibitor Cocktail Tablets (Merck) and homogenized three times for 30 s using the Precellys evolution (Bertin Technologies) bullet blender with 1.4 mm zirconium oxide Precellys bulk beads (Bertin Technologies). Homogenized tissue was further solubilized for 30 min at 4°C. Sample was centrifuged (800*g*, 10 min, 4°C) to remove the nuclear fraction and cell debris. The supernatant was centrifuged (maximum speed, 30 min, 4°C) and to maximize solubilization of membrane proteins the pellet was resolubilized in 500 µl of lysis buffer for 30 min. Insoluble proteins and debris were removed by centrifugation at maximum speed for 5 min and this fraction was added to the previously obtained supernatant. The supernatant was added to 500 µg of prewashed Pierce™ anti-HA magnetic beads (Invitrogen) and co-immunoprecipitated according to the manufacturer’s protocol. For a subset of trial experiments, Dynabeads™ co-immunoprecipitation kit (Invitrogen) was used in combination with a rabbit anti-V5 antibody (Ab3792, Millipore) instead. Here, 40 µg antibody was added to 1.5 mg beads per sample. For all experiments, bound protein complexes were gently eluted using 2× 50 µl of a 0.1 M glycine (pH = 2) solution by incubating for 10 min at room temperature. The solution was then neutralized with 15 µl of 1 M Tris (pH=8) and sent to the Centre for Proteomics (CfP, UA) together with the eluted beads and lysate fraction for mass spectrometric analysis of both fractions to maximize identification of immunoprecipitated proteins. Eluted fractions and lysates were reduced by addition of TCEP (1 h, 4°C) and alkylated with iodoacetamide (15 mM) for 20 min at room temperature in the dark before loading on S-trap micro columns (ProtiFi) following the manufacturer’s protocol, cleaved overnight with 1 µg trypsin at 37°C, dried with SpeedVac and stored at −80°C until mass spectrometry analysis. The bead fraction, containing beads with strongly bound proteins, was reduced for 1 h at 4°C after the addition of 50 mM triethylammonium bicarbonate solution (50 mM) with 5 mM TCEP, followed by 20 min alkylation in the dark (after the addition of 15 mM iodoacetamine). Next, 0.5 µg of trypsin was added followed by overnight cleavage at 37°C. The next day, the cleaved peptides were purified on C18 spin columns (Thermo Fisher) followed by speedvac drying and storage at −80°C until MS analysis.

Before MS analysis, samples were resuspended in Mobile Phase A and loaded on evotips for chromatographic separation using an Evosep One high-performance liquid chromatography system according to the manufacturer’s protocol. The Evosep One LC system was linked with the Bruker timsTOF Pro mass spectrometer, equipped with the Bruker Captive Spray source. The 30 SPD method was employed. The endurance column, measuring 15 cm × 150 μm ID with 1.9 μm beads (EV1106, Evosep), was connected to a Captive Spray emitter (ZDV) with a diameter of 20 μm (1865710, Bruker), both from Bruker Daltonik GmbH, Bremen. The timsTOF Pro underwent calibration in accordance with the manufacturer’s guidelines. Source parameters included a capillary voltage of 1500 V, dry gas at 3.0 l min^−1^, and dry temperature at 180°C. The ion transfer capillary was maintained at 180°C, while the column was held at 40°C. The Parallel Accumulation–Serial Fragmentation (PASEF) method was employed in data-dependent acquisition (DDA) mode to select precursor ions for fragmentation, involving 1 TIMS-MS scan and 10 PASEF MS/MS scans, as detailed by Meier *et al*. [65]. The TIMS-MS survey scan spanned 0.70–1.45 V s cm^−2^ and 100–1700 *m*/*z*, with a ramp time of 100 ms. The 10 PASEF scans comprised an average of 12 MS/MS scans per PASEF scan, using a collision energy of 10 eV. Precursors with 1–5 charges were selected with a target value set to 20 000 arbitrary units and an intensity threshold of 2500 arbitrary units. Precursors were dynamically excluded for 0.4 s. The OtofControl 6.0 software (Bruker Daltonik GmbH) controlled the timsTOF Pro. Ten PASEF scans could contain up to 12 MS/MS scans each. Data analysis used Peaks Xpro from Bioinformatics Solutions, Inc. (BSI, Ontario, Canada). Precursor mass tolerance was set at 20 ppm, fragment mass error tolerance at 0.05 Da. The enzyme was set to none, and the digest mode to unspecific. Oxidation (M) and deamidation (NQ) were specified as variable modifications. All samples were normalized and analysed using PEAKS (Bioinformatic Solutions, Inc). The selection of potential binding partners was done by analysing the ratio of area and spectra of HAV5/wild-type and pull-down/lysate. Protein binding partners were analysed via STRING [66].

### (o) Ultrasonic vocalizations

GPower analysis was used to calculate sample size, using the default alpha level of 5% and power of 80%. For USV experiments, effect size was estimated to be 0.7 based on previous publications, resulting in theoretical 34 animals per group. Female mice were mated with appropriate stud males and individually housed upon visible signs of pregnancy. Hereafter, cages were inspected daily for signs of birth. Isolation-induced pup USV were recorded on postnatal days P3, 6, 9 and 12 during the light cycle at room temperature. The number of pups used per genotype and age is shown in electronic supplementary material, table S1. To induce pup ultrasonic calling, pups were taken from their home cage and immediately placed into a glass container with fresh bedding positioned in a soundproof styrofoam box, applying a protocol previously established in the laboratory [16]. USV were recorded for a period of 10 min using the UltraSoundGate Condenser Microphone CM16 (Avisoft Bioacoustics) and transferred to the computer via the UltraSoundGate 116 USB audio device (Avisoft Bioacoustics) using Avisoft RECORDER software (Avisoft Bioacoustics). Following USV measurements, the surface body temperature of the pups was immediately measured using a surface temperature sensor and reader (Testo) placed on the pup’s stomach for 30 s. Hereafter, pup length and weight were measured. The pup was then placed back in a corner of the home cage to measure maternal retrieval time. In between pups, the glass container was thoroughly cleaned with 70% ethanol and fresh bedding was applied. At P3, after pup USV recording, a small amount of green Ketchum animal tattoo ink (Ketchum Manufacturing, Inc.) was injected into the paws of each pup for identification purposes, and a small piece of tail was taken for genotyping.

Recorded sound files were analysed using the SASlab Pro software (Avisoft Bioacoustics). First, a fast Fourier transform was conducted (512 FFT length, 100% frame, Hamming window, and 75% time window overlap) to create spectrograms with 488 Hz of frequency and 0.512 ms of time resolution. Next, section labels were generated using the ‘Automatic Parameter Measurements setup’. Here, the spectral entropy setting was used with a max entropy of 0.4 and a hold time of 30 ms. Identified elements were transferred into labels and each element was manually checked for overlap with the USV, after which the total number of USV was calculated. Peak frequency, duration, amplitude and frequency modulation were then measured at the mean of the element. The amplitude was measured in decibels relative to full-scale (dBFS) where 0 dBFS corresponds to the maximum level of the wave that can be recorded without clipping at the selected gain. All measured sound that does not surpass this is negative relative to this level. The absolute of the measured value in dBFS was then subtracted from 100.

A substantially large number of calls were collected from the ubiquitous and conditional KO pups over development. The values of all calls were averaged per pup and age, and in all cases, the numbers shown in electronic supplementary material, table S1, were used as sample size for the statistical analysis. For the comparison of peak frequency between genotypes, median values were used instead of averages, as the distribution is not Gaussian. Histograms of the average relative abundance of peak frequency per genotype, including 95% confidence intervals (dotted lines) were generated using GraphPad Prism. Owing to the variable number of animals used per experiment, a mixed-effects ANOVA with Geisser–Greenhouse correction and Tukey *post hoc* test was used unless otherwise noted. Kernel density estimation (KDE) plots were generated with Seaborn v0.12.2 [67] and matplotlib [68]. KDE values were scaled based on the number of data points in each dataset, and a common colour scale was determined using the minimum and maximum KDE values per feature. Using Plotly [69], interactive 3D scatter plots were generated for each age and genotype. Sequential correlation analysis of the duration of calls with previous calls (*N* − 1) was performed using Excel. Peak frequency, duration, amplitude and frequency modulation values were standardized and t-SNE was applied to the scaled data with a random state of 42 and a perplexity of 30. Scaled data were used for clustering via a spectral clustering approach using nearest neighbour affinity with a random state of 42. The characteristic parameters of each call cluster, along with the relative fractions of clusters throughout development or between genotypes were calculated using excel.

### (p) Analysis of single-cell RNA dataset

The dataset [70] was interrogated using the SeuratV3 R package [71]. Mitochondrial genes were removed using the ‘PercentageFeatureSet’ tool, and data were transformed using SCTransform. Data were normalized and scaled according to standardized procedures, and variable features (*n* = 3000, vst) were identified. To identify differential gene markers between ‘*Pcdh10* positive’ and ‘*Pcdh10* negative’ groups, novel metadata were added to all cells based on their *Pcdh10* count ( >0). By using the ‘FindAllMarkers’ tool of Seurat, differential genes with a fold change of at least 1.5 between both groups were identified. Gene Ontology (GO) over-representation analysis was performed with the ClusterProfiler package [72,73]. Gene symbols were converted to Entrez IDs using the MyGene package [74], and the Genome wide annotation for Mouse (‘org.Mm.eg.db’) database from BioConductor was used as background for the ‘enrichGO’ tool of ClusterProfiler. DotPlot was constructed via the ggplot2 package [75].

### (q) Statistical analysis

Statistical analysis was performed using GraphPad Prism 9 software (GraphPad Software). Appropriate statistical tests were used depending on the data distribution and experimental design. For normally distributed data, we employed *t*-tests or ANOVA followed by *post hoc* tests for multiple comparisons. Non-parametric tests such as the Mann–Whitney *U* test or Kruskal–Wallis test were used for non-normally distributed data. In all cases, a *p*-value less than 0.05 was considered statistically significant. Data are presented as mean ± s.d. unless otherwise noted.

## 3. Results

### (a) Spatial and temporal dynamics of *Pcdh10* expression in the developing brain

**Figure 1.**
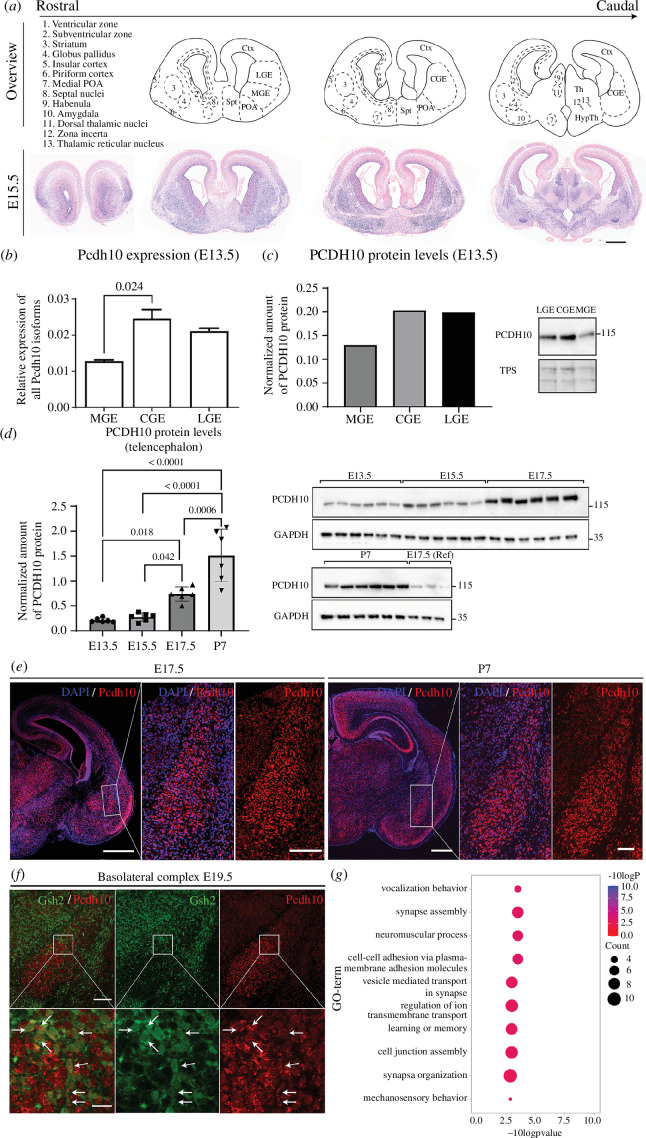
*Pcdh10* in the embryonic and juvenile murine wild-type brain. (*a*) Overview of *in situ* hybridization against murine *Pcdh10* on coronal sections at E15.5. Scale bar, 500 µm. (*b*) Relative expression of *Pcdh10* in the GEs at E13.5. *Pcdh10* levels are highest in CGE and LGE. Expression is normalized with housekeeping genes Gapdh and B-akt. *p = 0.024 as assessed with one-way ANOVA with Tukey *post hoc* test. *n* = 2. (*c*) Quantification of relative protein levels of PCDH10 in pooled samples of the GEs at E13.5 and corresponding blot showing the protein levels of PCDH10 and a TPS in pooled samples of the GEs at E13.5. Samples are a mix of three biological replicates. *n* = 1. (*d*) Quantification of relative protein levels of PCDH10 in the telencephalon and corresponding blot showing the protein levels of PCDH10 and GAPDH in the telencephalon at different stages of development (E13.5, E15.5, E17.5, P7). Protein levels at P7, located on a separate blot, were normalized with reference (Ref) E17.5 values, present on both blots, to address inter-blot variability. Protein levels increase over embryonic and postnatal development. Protein levels were normalized with GAPDH. *p_E13.5–E17.5_ = 0.018, *p_E15.5–E17.5_ = 0.042, ***p_E17.5–P7_ = 0.0006, ****p_E13.5–P7_ and *p*_E15.5–P7_ ≤ 0.0001, as assessed with one-way ANOVA with Tukey *post hoc* test. *n* = 6. (*e*) Overview of coronal paraffin brain sections (E17.5 (left), P7 (right)) stained with hybridization chain reaction against murine *Pcdh10*. Scale bar, 500 µm. Zoom-in on BLC on coronal brain sections is shown. Scale bar, 100 µm. (*f*) Overview of the BLC on coronal sections with *Gsh2*-lineage labelled green fluorescent and stained with HCR probes against *Pcdh10* (E19.5). *Pcdh10* is present in both excitatory and inhibitory neurons. Scale bar, 100 µm. Zoom in is shown on bottom. Pinhole, 85 µm. Scale bar, 20 µm. (*g*) Dotplot visualizing the top 10 over-represented GO terms for the ‘biological process’ group for genes identified for the ‘*Pcdh10* positive’ and ‘*Pcdh10* negative’ groups in adult murine brain (P56). Ctx, Cortex; LGE, lateral ganglionic eminence; MGE, medial ganglionic eminence; CGE, caudal ganglionic eminence; POA, pre-optic area; Spt, septum; Th, thalamus; HypTh, hypothalamus.

According to our hypothesis, *Pcdh10* might be involved in interneuron development and maturation. To get a better overview of *Pcdh10* expression at earlier developmental stages, *Pcdh10* mRNA was investigated on wild-type (Wt) embryonic (E15.5) coronal paraffin sections using ISH (figure 1*a*). At this stage, expression can predominantly be found in the subpallium and the ventricular zone (VZ) of the pallium. Within the subpallium, *Pcdh10* was expressed in the VZ and mantle zone (MZ) of the GEs, but a clear signal was missing from the subventricular zone (SVZ). Moreover, a salt-and-pepper expression pattern was present, with regions of increased expression in the telencephalon including the striatal region, the insular cortex, the bed nucleus of the stria terminalis, the piriform cortex, the lateral preoptic area (POA) and the amygdala, in particular in the nucleus of the lateral olfactory tract (nLOT). The migratory stream from the pallium towards the amygdala (the ventropallial migratory stream (VMS)) outlined a region with strong *Pcdh10* expression within the subpallium. In the thalamus, the habenula and dorsal thalamic nuclei are clearly marked, in addition to the zona incerta (ZI) and the thalamic reticular nucleus (TRN).

The GEs generate the majority of interneurons in the telencephalon, a cell type that is implicated in ASD aetiology. Progenitor cells from the GEs give rise to various subtypes of interneurons, each programmed to mature into interneurons with unique functions and destinations. To investigate whether *Pcdh10* is preferentially expressed in the different GEs, medial (MGE), lateral (LGE) and caudal (CGE) GE tissue was dissected at E13.5 and the mRNA content was analysed. As shown in figure 1*b*, normalized expression of *Pcdh10* was highest in the CGE, closely followed by LGE and lowest in MGE. At E13.5, protein levels of PCDH10 in the GEs follow a similar pattern to mRNA expression (figure 1*c*). To study the dynamics of PCDH10 protein levels during embryonic brain development, and potentially link *Pcdh10* to developmental processes ongoing at different ages, whole telencephalon was collected at E13.5, E15.5, E17.5 and P7 and submitted to western blot analysis (figure 1*d*). PCDH10 levels were found to rise, first steadily at mid-to-late gestational stages (E13.5–E17.5), then sharply between late embryonic stages (E17.5) and early postnatal life (P7), hinting towards a strong involvement in of *Pcdh10* in postnatal development (*p* = 0.979 (E13.5–E15.5), *p* = 0.018 (E13.5–E17.5), *p* < 0.0001 (E13.5–P7), *p* = 0.042 (E15.5–E17.5), *p* < 0.0001 (E15.5–P7), *p* = 0.0006 (E17.5–P7), *n* = 6). The original western blot images are shown in electronic supplementary material, figure S2.

Previous studies show strong *Pcdh10* expression in the juvenile and adult amygdaloid complex. Our ISH results at E15.5 imply that this expression already emerges during embryonic development. We further investigated this expression at late embryonic and early postnatal developmental stages with HCR (figure 1*e*). At both E17.5 and P7, the BLC can be delineated within the basal forebrain based on the strong signal of *Pcdh10.* At P7, *Pcdh10* is strongly expressed in the basolateral amygdala, with a weak, patchy expression pattern visible in the lateral amygdala. This difference in expression levels is already noticeable at embryonic stages (E17.5) but less pronounced. The BLC of the amygdala is primarily referred to as a pallial- or cortical-like area within the ventral forebrain, with the majority of neurons being excitatory neurons derived from the pallium, and a lower percentage of subpallium-derived inhibitory interneurons (basal amygdala: 22%, lateral amygdala: 16%) [76]. To investigate the expression of *Pcdh10* in *Gsh2*-lineage interneurons of the BLC, an HCR was performed at E19.5 in brains in which the *Gsh2*-lineage was labelled with eGFP. *Pcdh10* was expressed both in *Gsh2-*lineage interneurons (white arrows), as well as in other, likely excitatory, cells of the BLC (figure 1*f*).

To gain a first unbiased view on the cell-type specificity and the potential role of cells expressing *Pcdh10* in the mouse brain, a published scRNAseq adult mouse telencephalon dataset was analysed [70]. *Pcdh10* was moderately expressed in the cells, with 27.5% of all cells containing at least one count of *Pcdh10*. A method based on virtual differential gene expression analysis via the Seurat function ‘FindAllMarkers’ was then performed. Here, we identified genes that could be considered markers for the group of cells that show at least one *Pcdh10* count versus the group of cells that do not show any counts for *Pcdh10* (cut-off > 0). Forty-nine differentially expressed genes with a fold change of at least 1.5 were identified using this method (electronic supplementary material, table S2). As shown in figure 1*g*, highly relevant significant GO terms of biological process (BP) were identified. The GO term ‘vocalization behaviour’ is associated with ASD and links to the observed phenotype of *Pcdh10* mutant mice [34,36]. This implies that *Pcdh10* is expressed in cell types involved in the control of speech or, alternatively, can be found in a common regulatory network shared with other vocalization-related genes (*Foxp2, Cntnap2, Nrxn1, Nrxn3*). Among the identified markers several genes that are linked to ASD are present, including two neurexins (*Nrxn1 and Nrxn3*), the receptor-type tyrosine-protein phosphatase T gene (*Ptprt*) and the vocalization gene contactin-associated protein 2 (*Cntnap2*). In addition to vocalization and ASD, multiple over-represented GO terms congregate at the synaptic level.

### (b) PCDH10 binds synaptic proteins at postnatal day 7

**Figure 2.**
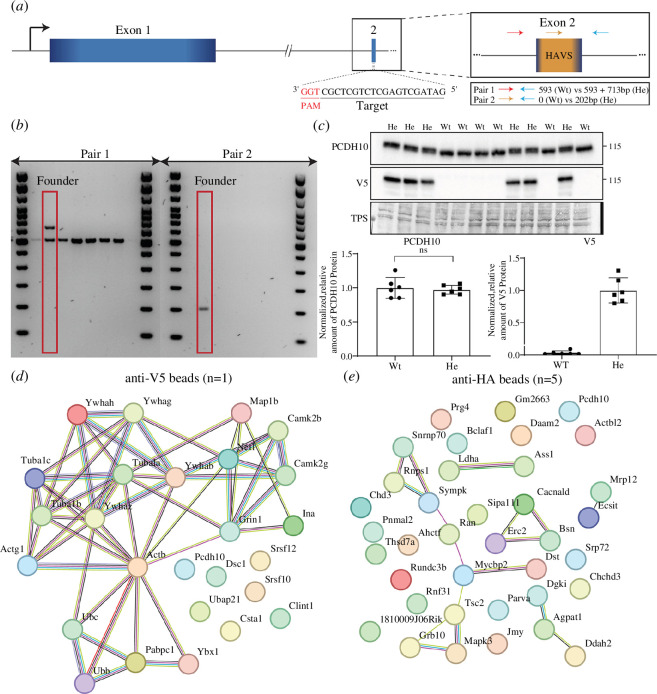
Binding partners of PCDH10 congregate at the synaptic level. (*a*) Visual representation of the location and sequence of selected guide RNA targeting long isoform of *Pcdh10*. Zoom-in on targeted exon 2 is shown, including a PCR strategy to discriminate between successfully edited and wild-type pups. The location of primers is schematically shown. Primer pair 1 (red–blue) surrounds the insertion. Forward primer of primer pair 2 (yellow–blue) binds the to be inserted sequence and only results in the generation of an amplicon upon successful integration. (*b*) Gel electrophoresis result of a PCR on DNA from a portion of pups born from iGONAD-treated females, amplified with both primer pairs shown in (*a*). Red boxes highlight the potential founder. (*c*) Quantification and blots showing the protein levels of PCDH10 and V5 in the *Pcdh10-*HAV5 telencephalon (E17.5). V5 protein could only be detected in heterozygous samples, while no significant differences in PCDH10 protein level could be detected between heterozygous and wild-type mice. Protein levels are normalized with a TPS and relative to heterozygous levels. *p* > 0.05 as assessed by two-tailed *t*‐test. *n* = 6. Wt, wild-type, He heterozygous. (*d*) STRING network of identified potential binding partners of PCDH10 in a Co-IP trial experiment, using anti-V5 beads. Identified partners are strongly related to the pre- and post-synaptic compartment and the cytoskeleton. *n* = 1, technical replicates = 3. (*e*) STRING network of identified potential binding partners of PCDH10 from a co-immunoprecipitation experiment, using anti-HA beads. Identified proteins are related to the pre- and post-synapse and the cytoskeleton. *n* = 5.

To gain further insight into the molecular mechanism of PCDH10 function in the developing brain, a novel epitope-tagged *Pcdh10* mouse line for use in co-immunoprecipitation (Co-IP) experiments was generated. C-terminal tagging was impossible owing to the expression of different *Pcdh10* isoforms in the developing brain, which each contain a unique C-terminus (electronic supplementary material, figure S3A). These isoforms are all expressed in the GEs at E13.5 (electronic supplementary material, figure S3B). Likewise, N-terminal tagging is hindered by the presence of a signal peptide in all isoforms. Instead, a combination of structural analysis and bioinformatics tools was used to predict solvent accessibility, secondary structures and conserved, functional and/or possible protein binding regions within PCDH10 (PredictProtein, PsiPred, ModPepInt, pSort II) [77–80]. We identified a possible tagging region at the beginning of exon two, in the cytoplasmic tail, that was accessible to solvents, had a low conservation score and was located in a region that is shared by all long isoforms (electronic supplementary material, figure S3C). A CRISPR guide RNA was designed that targeted this region, together with a repair template containing the sequence of our epitope of choice (HAV5), linkers and flanking homology arms to allow homology-directed repair (figure 2*a* and sequence repair template; see table 2).

The iGONAD protocol [54,55] was used to generate *Pcdh10* knock-in mice. An optimized procedure was applied in combination with the gRNA targeting exon 2 and the repair template to generate knock-in *Pcdh10*-HAV5 mice. Three genotyping primers were designed with the first pair surrounding the insertion site, so that the amplicon could be discriminated from wild-type DNA by amplicon length, while the forward primer of the second pair targeted a sequence within the HAV5 insertion. As a result, the amplicon could only be generated after a successful knock-in (figure 2*a*,*b*). Based on gel electrophoresis of the generated amplicons, a potential founder was selected (figure 2*b*). Sequencing results confirmed a heterozygous insertion of the HAV5 sequence. To investigate whether the insertion site was accessible to antibody, and whether the insertion had a detrimental effect on PCDH10 protein levels, a western blot was carried out on the telencephalon of E17.5 *Pcdh10-*HAV5 mice. As shown in figure 2*c*, V5 protein was detected in the heterozygous mice; moreover, the tagging had no discernible effects on PCDH10 protein levels.

To optimize the Co-IP procedure on P7 *Pcdh10*-HAV5 telencephalon tissue, a trial experiment was performed. First, the membrane fraction with and without crosslinking with PFA (0.1%) was pulled down using anti-V5 beads and analysed with mass spectrometry (one biological sample per condition, three different technical conditions). Several interesting proteins were identified, visualized in the STRING network in figure 2*d*, including NMDA-subunit GRIN1 and CAMKII β and γ subunits. Enriched GO terms of BP included peripheral nervous system axon regeneration, post-synaptic cytoskeleton organization, neurofilament cytoskeleton organization and protein localization to cell–cell junction. Molecular function (MF) GO terms included structural constituent of post-synaptic actin cytoskeleton, structural constituent of post-synaptic intermediate filament, structural constituent of post-synapse, protein tag (Ubi) and structural constituent of cytoskeleton. Finally, cellular components included post-synaptic intermediate filament cytoskeleton, calcium- and calmodulin-dependent protein kinase complex, dense body, post-synaptic cytoskeleton and presynaptic cytoskeleton. Although this experiment yielded interesting preliminary results, a switch from the anti-V5 beads to anti-HA beads was made to increase elution efficiency. Moreover, the trial experiment showed that the PFA condition was able to enrich the captured interaction partners. Consequently, a second experiment was conducted solely using lightly fixated samples (*n* = 5). Potential interaction partners were selected based on several criteria; known contaminations were removed, the ratio of the sum of the area of HAV5 samples versus sum of wild-type samples needed to be sufficiently high (>5) or the sum of the spectra of HAV5 samples versus sum of wild-type samples needed to be sufficiently high (>5). Proteins that qualified for these criteria were further sorted based on the enrichment of the ratio of pull-down versus lysate of HAV5 samples and the amount of HAV5 samples that contained the identified protein. As shown in the string network in figure 2*e*, several potential interaction partners were identified, including CACNA1D, BSN, MYCBP2, TSC2, ERC2, RAN GTPase and DGKι. Enriched GO terms include actin filament-based process (BP), cytoskeleton (Cellular Compartment (CC)) and Phospholipase D signalling pathway (MF). Identified subcellular compartments (via STRING) include the cytoskeleton of the presynaptic active zone, presynaptic active zone, post-synaptic density and plasma membrane-bounded cell projection. Although these binding partners currently remain preliminary and unvalidated, these data together with previous published studies and the scRNA dataset strongly point toward a role of PCDH10 at the synapse in the developing and adult brain (P7).

### (c) Conditional loss of Pcdh10 affects Gsh2-lineage interneuron numbers in the basolateral complex

**Figure 3.**
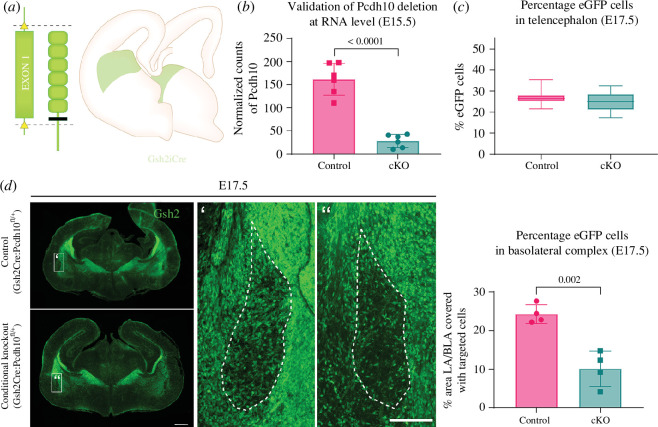
Reduced amount of *Gsh2*-lineage interneurons in the BLC (E17.5) in a *Pcdh10* conditional KO model. (*a*) Visual representation of the *Pcdh10* conditional KO model. The first floxed exon of *Pcdh10* is excised in targeted cells after crossing with a Cre-driver line. An additional crossing is done with RCE mice to induce green fluorescence in targeted cells. (*b*) Validation of the *Pcdh10* conditional KO model via bulk RNA sequencing of control and conditional KO telencephalon at E15.5. Counts are normalized with size factors calculated by DESeq2 and significantly reduced in conditional KO mice as compared to control mice. ****p < 0.0001 as assessed with two-tailed unpaired *t*‐test. *n* = 6. (*c*) Percentage targeted (eGFP^+^) cells in the telencephalon of control and conditional KO embryos at E17.5. No significant differences could be observed as assessed with two-tailed unpaired *t*‐test, *p* > 0.05. *n* = 11 (Control) and *n* = 6 (cKO). (*d*) Overview of *Gsh2* expression in coronal vibratome sections (60 µm) of control and conditional KO telencephalon at a caudal level (E17.5). Scale bar, 500 µm. Zoom-in on the BLC is shown (scale bar, 100 µm). Quantification of the percentage of BLC area that is occupied by targeted *Gsh2*-lineage interneurons in control and conditional KO embryos (E17.5). Targeted (eGFP^+^) interneurons are reduced in the BLC of the conditional KO as compared to the control condition. A significant difference is present between control and conditional knockout, as assessed with two-tailed unpaired *t*‐test. **p = 0.002. *n* = 4.

To investigate whether interneurons are involved in the phenotypes previously observed in *Pcdh10* mutant mice, we specifically knocked out *Pcdh10* in interneurons, by crossing *Pcdh10* ^fl/+^ mice [50] with *Gsh2*-iCre transgenic mice [52] (figure 3*a*). *Gsh2* emerges early during embryonic development (E9.5), selectively labels LGE- and part of CGE-derived interneurons in the telencephalon, and delivers cells eventually populating many of the regions with high *Pcdh10* expression, including the amygdala, striatum and olfactory bulbs [81–84]. By crossing this line with a transgenic floxed RCE line [51], all *Gsh2-*expressing cells and their progeny will be green fluorescent. The successful KO of *Pcdh10* in *Gsh2*-lineage cells was validated in sorted, targeted cells (eGFP^+^) via bulk sequencing at E15.5, and a strong reduction of *Pcdh10* counts could be observed in the telencephalon of conditional KO (cKO) embryos as compared to control embryos (control: 161.60 ± 34.58, cKO: 28.39 ± 14.12, *p* < 0.0001, *n* = 6, figure 3*b*).

Interneuron dysfunction can contribute to the development of ASD, and *Pcdh10* shows an exceptionally strong expression in the subpallium. A crucial step in elucidating the role of *Pcdh10* in brain development and ASD was to investigate the positioning and amount of interneurons in the Gsh2:cKO model. We analysed the amount of targeted (*Gsh2*-lineage, eGFP^+^) cells in the entire telencephalon of Gsh2:Control and Gsh2:cKO embryos with flow cytometry at E17.5 (figure 3*c*). No significant difference could be observed in the percentage of targeted cells between cKO and Control (*p* = 0.492). In addition, no broad abnormal positioning of the interneurons could be observed within this model. However, specifically within the BLC, the area occupied by targeted *Gsh2*-lineage interneurons was strongly reduced in cKO brain as compared to control brains (control: 24.27% ± 2.44, cKO: 10.12% ± 4.61, *p* = 0.002, *n* = 4, figure 3*d*). Since no difference was observed in the total amount of targeted cells, this reduced signal in the BLC is not a result of overall reduced *Gsh2-*lineage interneurons in the telencephalon.

### (d) Pcdh10 deficiency leads to increased emission of isolation-induced ultrasonic vocalizations

**Figure 4.**
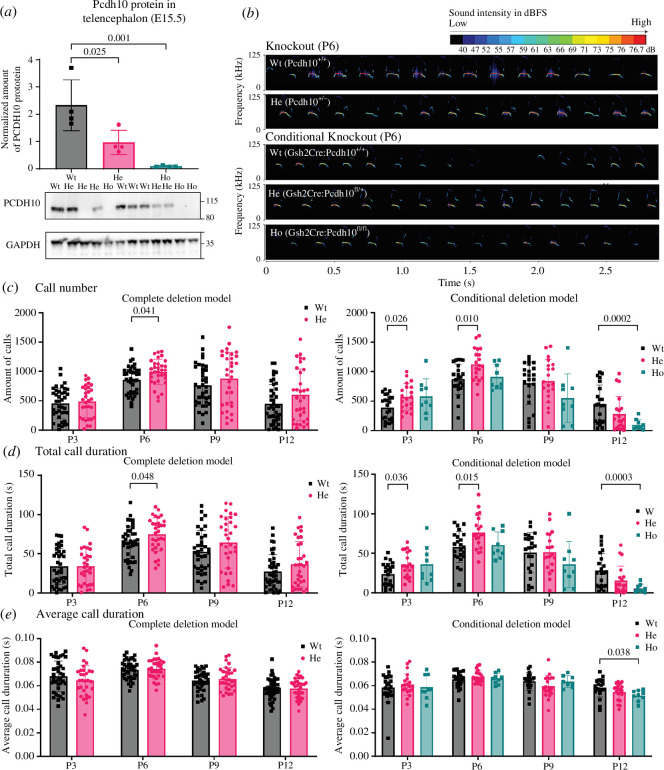
Abnormal USV in heterozygous and homozygous *Pcdh10* KO pups between P3 and P12. (*a*) Validation of *Pcdh10* KO line via western blot (P7). The amount of protein is significantly reduced in heterozygous and homozygous pups, *p_Wt–He_ = 0.025 and *** *p*_Wt–Ho_ = 0.001 as assessed with one-way ANOVA. *n* = 4. (*b*) Representative spectrograms of USV of a P6 wild-type, heterozygous *Pcdh10*KO, and heterozygous and homozygous conditional *Pcdh10* KO littermates. (*c*) Amount of isolation-induced USV emitted by wild-type and heterozygous *Pcdh10*KO pups and by wild-type, heterozygous and homozygous *Gsh2*-lineage *Pcdh10* conditional KO pups. USV of heterozygous KO are increased at P6, *p KO P6 = 0.041 as assessed with a mixed-effects two-way ANOVA with Holm–Sidak *post hoc* test. USV of heterozygous cKO pups are increased at P3 and P6 as compared to wild-type pups, while USV of homozygous cKO pups are decreased at P12 as compared to wild-type pups. *p_P3 Wt–He_ = 0.026, **p_P6 Wt–He_ = 0.010, ***p_P12 Wt–Ho_ = 0.0002 as assessed with a mixed-effects two-way ANOVA with Tukey *post hoc* test. (*d*) Total call duration of isolation-induced USV in wild-type and heterozygous Pcdh10KO pups and in wild-type, heterozygous and homozygous *Gsh2*-lineage *Pcdh10* conditional KO pups. Total call duration of heterozygous KO pups is increased at P6, *p P6 Wt–He = 0.048 as assessed with a mixed-effects two-way ANOVA with Holm–Sidak *post hoc* test. Total call duration of heterozygous cKO pups is increased at P3 and P6 as compared to wild-type pups. Total call duration of the USV of homozygous cKO pups is decreased at P12 as compared to wild-type pups. *p_P3 Wt–He_ = 0.036, *p_P6 Wt– He_ = 0.015 and ***p_P12 Wt–Ho_ = 0.0003 as assessed with a mixed-effects two-way ANOVA with Tukey *post hoc* test. (*e*) Call duration of isolation-induced USV in wild-type and heterozygous *Pcdh10*KO pups and in wild-type, heterozygous and homozygous *Gsh2*-lineage *Pcdh10* conditional KO pups. Call duration of heterozygous *Pcdh10*KO pups is similar to wild-type pups and call duration of homozygous cKO pups is decreased at P12 as compared to wild-type pups. *p_cKO P12 Wt–Ho_ = 0.038 as assessed with a mixed-effects two-way ANOVA with Tukey *post hoc* test.

Previous studies indicated that mice haploinsufficient for *Pcdh10* displayed alterations in socio-affective communication through isolation-induced USV [34,36], a change typically associated with increased anxiety in the pup [85,86]. These studies however did not investigate which cells might be responsible for this phenotype, and did not distinguish between potential cell type-specific effects of *Pcdh10* haploinsufficiency. A KO model and the Gsh2:cKO model were therefore compared to investigate whether altered *Pcdh10* expression in interneurons affects ultrasonic calling, as a measurement for anxiety. To mitigate the effect of the LacZ cassette in the mouse line used by Schoch *et al*. [36], a new ubiquitous *Pcdh10* KO line was generated. To this end, male *Pcdh10* ^fl/+^ mice were crossed with a general maternal Cre line (*Pgk1*-Cre) to target all cells of the developing progeny [53]. After backcrossing for at least three generations, protein was isolated and the loss of PCDH10 was validated in whole body of heterozygous (He) and homozygous (Ho) embryos (E17.5). Herozygous embryos contained halved PCDH10 levels and homozygous embryos completely lost PCDH10 within the telencephalon (Ho: 0.089 ± 0.031, He: 0.963 ± 0.444, Wt: 2.328 ± 0.935, *p* = 0.025 (Wt–He), *p* = 0.001 (Wt–Ho), *p* = 0.152 (He–Ho), one-way ANOVA, *n* = 4, figure 4*a*). The original western blot is again shown in electronic supplementary material, figure S2.

Pups of both lines were monitored for 15 days following birth and all pups showed normal weight gain, growth and body temperatures (electronic supplementary material, figure S4). Development but not genotype contributed to changes in these parameters (ageing *p* < 0.0001). Isolation-induced USV were measured every third day from P3 to P12 and a representative spectrogram of a P6 *Pcdh10* ^+/+^ (KO Wt), *Pcdh10* ^+/−^ (KO He), *Gsh2*Cre^+/+^:*Pcdh10* ^X/X^ (Gsh2:cKO Wt), *Gsh2*Cre^+/−^:*Pcdh10* ^+/fl^ (Gsh2:cKO He) and *Gsh2*Cre^+/−^:*Pcdh10* ^fl/fl^ (Gsh2:cKO Ho) pup is shown in figure 4*b*. Across test days, more than 300 000 isolation-induced USV were recorded and analysed. The total call number was assessed for all lines and genotypes (figure 4*c*). In the *Pcdh10* KO line, genotype affected USV numbers (*p* = 0.037). Specifically, pups haploinsufficient for *Pcdh10* (ubiquitous KO) showed increased USV emission rates as compared to their wild-type littermates, significant at P6 when these calls peak and persisting until P12 (P6: *p* = 0.041). No developmental shift in the period of USV production could be identified (*p* age × genotype > 0.05). In contrast, the developmental trajectories of USV numbers differed between genotypes for the cKO line (*p* age × genotype < 0.0001). Similar to haploinsufficient KO pups, heterozygous cKO pups showed increased levels of isolation-induced USV as compared to wild-type littermates, observable as early as P3 and peaking at P6 (P3: *p* = 0.026; P6 *p* = 0.010). At these ages, the difference between heterozygous cKO pups and wild-type littermates even surpassed those observed between heterozygous *Pcdh10* KO pups and controls. By P9, this difference became less pronounced, levelling off to wild-type levels by P12. Notably, call numbers from homozygous and heterozygous cKO pups were comparable at P3, but values from homozygous pups thereafter decreased, resulting in comparable numbers to wild-type pups at P6 and decreased numbers at P9 and P12, respectively (P12: *p* = 0.0002 (Wt–Ho)). Total call duration revealed similar trends for the ubiquitous and conditional *Pcdh10* KO mice (figure 4*d*). In general, call duration changed throughout development but was unaffected by genotype, although the developmental trajectory of the duration of calls differed between genotypes of the cKO (age *p* < 0.0001, cKO age × genotype *p* = 0.0001, figure 4*e*). In addition to the reduced number of calls between wild-type and homozygous cKO pups at P12, call duration was decreased (*p* = 0.038 (Wt–Ho)).

### (e) Acoustic call features of isolation-induced ultrasonic vocalizations in conditional Pcdh10 KO mice

**Figure 5.**
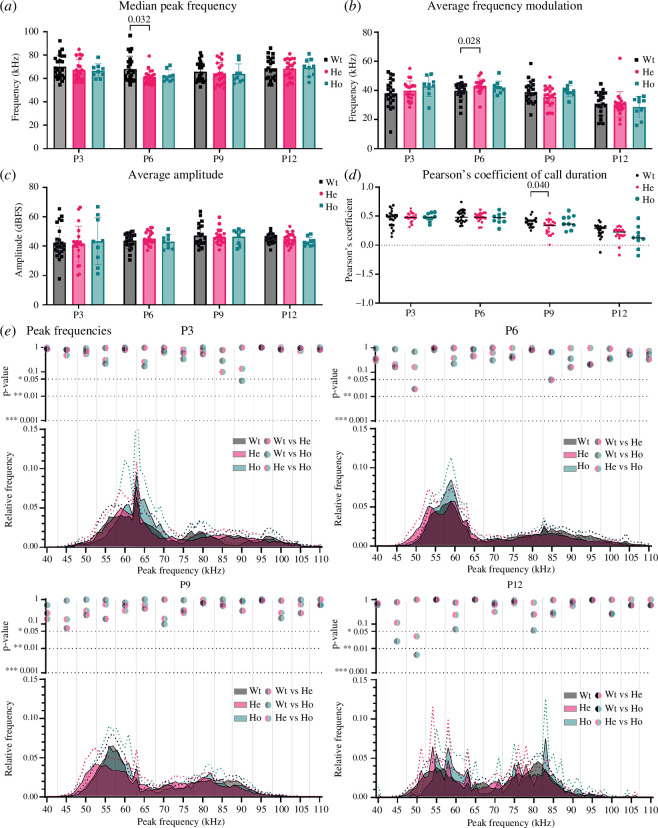
*Pcdh10* conditional KO pups exhibit a shift in peak frequency and amplitude in USV. (*a*) The peak frequency, (*b*) frequency modulation and (*c*) amplitude of isolation-induced USV in wild-type, heterozygous and homozygous *Gsh2*-lineage *Pcdh10* conditional KO pups. Peak frequency of the USV of heterozygous pups is decreased at P6 as compared to wild-type pups, while frequency modulation is increased. *p_P6 median peak frequency Wt–He_ = 0.032 and *p_P6 frequency modulation Wt–He_ = 0.028 as assessed with a mixed-effects two-way ANOVA with Tukey *post hoc* test. USV amplitude is unchanged between genotypes. (*d*) Pearson’s coefficient of call duration (*N* − 1) of isolation-induced USV in wild-type, heterozygous and homozygous *Gsh2*-lineage *Pcdh10* conditional KO pups. Correlation reduces throughout development. Pearson’s coefficient is significantly reduced in heterozygous pups as compared to wild-type littermates at P9. *p_P9 Wt–He_ = 0.040 as assessed with a mixed-effects two-way ANOVA with Tukey *post hoc* test. Dotted line corresponds to a correlation coefficient of 0. (*e*) Histograms showing the relative fraction versus peak frequency of isolation-induced USV in wild-type, heterozygous and homozygous *Gsh2*-lineage *Pcdh10* conditional knockout. The *p*-values of the grouped relative frequency bins are shown. Dotted lines correspond to *p*-values of 0.05 (*), 0.01 (**) and 0.001 (**). *p ≤ 0.05, **p ≤ 0.01, ***p ≤ 0.001. Significance was determined with a mixed-effects two-way ANOVA with Tukey *post hoc* test.

Previous studies revealed that differences in USV emission rates and acoustic call features, including amplitude, peak frequency and frequency modulation, can affect maternal retrieval behaviour [13]. Owing to the increased specificity of the experimental manipulation and the more prominent effect of *Pcdh10* deletion in *Gsh2-*lineage interneurons on isolation-induced USV, we opted to further investigate acoustic call features in the conditional *Pcdh10* KO line. Peak frequency, call duration and frequency modulation all changed throughout development (age *p*-value for peak frequency *p* = 0.0008, call duration *p* < 0.0001, frequency modulation *p* < 0.0001). At P6, calls were emitted at lower median peak frequencies in heterozygous as compared to wild-type pups (*p* = 0.032, figure 5*a*), while the frequency modulation was increased (*p* = 0.028 (Wt–He), figure 5*b*). The amplitude of calls was unchanged between genotypes (figure 5*c*). Moreover, sequential analysis of the call duration revealed that there is a positive correlation between each call (*N*) and the previous call (*N* – 1) (figure 5*d*). This correlation was relatively high at P3 and P6 (*r* > 0.4) and then decreased as development progressed, suggesting a high level of stereotypic call emission at early developmental stages. The developmental progression of these correlation values is affected by genotype in the conditional KO (*p* age × genotype = 0.041). Heterozygous cKO pups exhibit a steeper decrease of correlation values over development as compared to wild-type littermates from P9 onward, potentially affecting their ability to communicate with their mother or littermates (P9 *p* = 0.040 (Wt–He)).

Different call types have previously been identified in isolated rat and mouse pups. To classify the different types of isolation-induced USV, we further analysed the more than 130 000 isolation-induced calls emitted by the wild-type, heterozygous and homozygous *Pcdh10* conditional KO pups over their development. Histograms with binned *p*-values of the relative fraction of peak frequency values averaged by pup were created and analysed for each age and genotype (figure 5*e*). This allowed us to observe differences that were not evident from purely analysing averaged or median values, as peak frequency is not normally distributed. As shown, two main bands of peak frequency can be observed throughout development, including one band around 60 kHz (55–65 kHz) and one broader band around 80 kHz (70–100 kHz), from now on referred to as low- and high-pitch USV. Over development, the peak frequency of both bands decreased and the spread of values in both bands became less prominent. In addition, the relative contribution of the high-pitch calls (~80 kHz) increased with age. When comparing wild-type, heterozygous and homozygous conditional KO pups, subtle differences in peak frequencies can be observed. The relative contribution of the low-pitch calls is slightly increased for the homozygous cKO pups at earlier stages (P3–P6), but normalizes after the first postnatal week. Visible from P6 onward is the slight decrease of the peak frequency of calls emitted by heterozygous cKO pups as compared to wild-type and homozygous littermates, significantly different for the 47.5–52.5 kHz bin (*p* = 0.020) and 82.5–87.5 kHz bin (*p* = 0.049). This difference is still visible at P9, but not significant, and has disappeared by P12.

**Figure 6.**
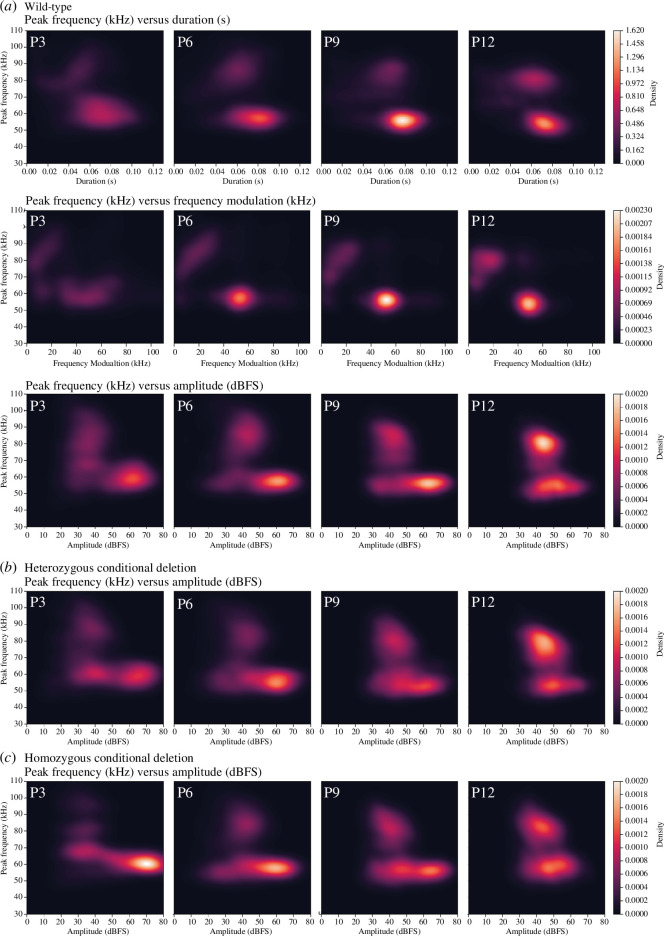
Call clusters change throughout development and between conditional *Pcdh10* KO genotypes. (*a*) Kernel density estimation plots of peak frequency (kHz) versus duration (s), frequency modulation (kHz) and amplitude (dBFS) of wild-type pups throughout development at postnatal days (P)3, P6, P9 and P12. Pattern and density of call clusters change throughout development. (*b*,*c*) Comparison of kernel density estimation plots with peak frequency (kHz) versus amplitude (dBFS) showing identified call clusters and densities of *Gsh2*-lineage conditional KO heterozygous (He, *b*) and homozygous (Ho, *c*) pups at postnatal days (P)3, P6, P9 and P12.

To better understand the distribution of calls with certain peak frequency, duration, amplitude and frequency modulation values over development and between genotypes, kernel density plots and three-dimensional interactive plots were generated (figure 6 and interactive electronic supplementary material). In figure 6*a*, wild-type profiles of peak frequency versus the other parameters are shown from P3 to P12. Clearly visible in all plots is the previously described emergence of high-pitch calls as the pups age, evolving from a diffuse cluster to a more distinct cluster (top half of figure 6*a*). In general, the range of the duration of calls decreased as development progressed (P3: 0.010.11 s; P12: 0.03–0.10 s). Interestingly, the frequency modulation of calls at early postnatal stages (P3) is distributed across a large range (0−80 kHz) but evolves towards two major groups of frequency modulation at later stages (P6 onward, 0−30 kHz and 40−65 kHz). Similar to the duration, the variation in amplitude across all calls becomes less extreme over time, with the majority of calls situating between 20 and 80 dBFS at P3 and between 30 and 70 dBFS at P12.

The two call types defined by their peak frequency each show their own preferential duration, amplitude and frequency modulation. In general, low-pitch calls show a slightly increased duration and are louder as compared to high-pitch calls (figure 6*a* and interactive electronic supplementary material). Moreover, low-pitch calls show a medium-to-high frequency modulation, evident from P6 onward when frequency modulation becomes more refined (40–65 kHz). In contrast, the majority of high-pitch calls have low-frequency modulation values at all ages (0–30 kHz).

When comparing between genotypes, call amplitudes show a shift towards louder calls in the homozygous cKO pups at P3 (figure 6*b*,*c* and interactive electronic supplementary material). Likewise, we observe a subtle relative increase of calls at lower peak frequency, together with a relatively decreased amount at the higher frequency band in the homozygous condition, with the heterozygous condition showing an intermediate state (electronic supplementary material, figure S5). No overt differences in the call duration were observed in KDE plots of all genotypes (electronic supplementary material, figure S5A). Notably, in the homozygous condition at P3 high-pitch calls show further separation based on peak frequency, with a band around 80 kHz and a band around 100 kHz instead of the more continuous peak frequency range in the heterozygous and wild-type condition (electronic supplementary material, figure S5B, interactive files).

### (f) Call cluster analysis of isolation-induced ultrasonic vocalizations

**Figure 7.**
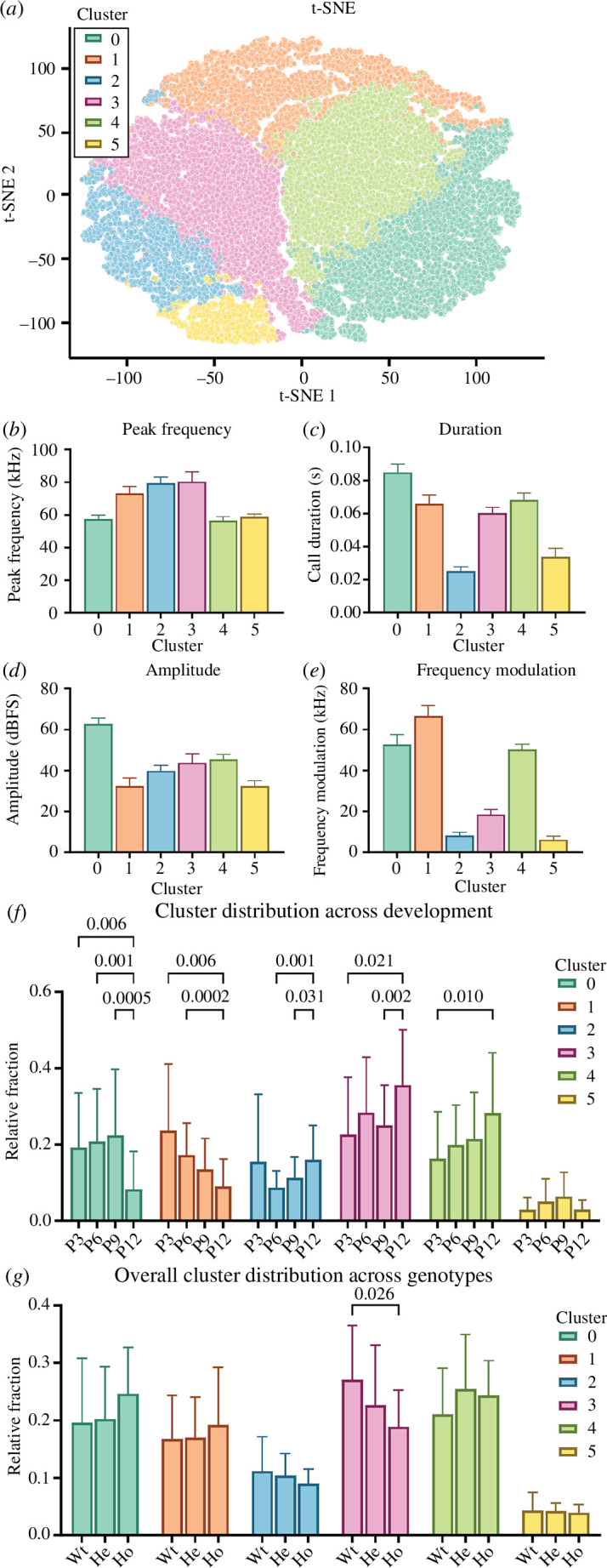
Dynamic appearance of different call types throughout development and impact of conditional *Pcdh10* loss. (*a*) *t*-SNE plot of all calls labelled by their respective cluster. (*b−e*) Average peak frequency (*b*)*,* duration (*c*)*,* amplitude (*d*) and frequency modulation ( *e*) values per cluster. Significance was determined with a one-way ANOVA with Tukey *post hoc* test for (*b–e*)*.* (*f*) The relative fraction of wild-type calls belonging to a cluster over development (P3–P6–P9–P12)*. p-*Values were determined with a mixed-effects two-way ANOVA with Tukey *post hoc* test. (*g*) The relative fraction of calls from wild-type, heterozygous and homozygous conditional KO pups per cluster at all ages. *p_cluster 3 Wt–Ho_ = 0.026 as assessed with a mixed-effects two-way ANOVA with Tukey *post hoc* test.

To better illustrate the existence of different call types, calls were clustered into six clusters via a spectral clustering algorithm. The t-SNE plot and the average cluster values for peak frequency, call duration, amplitude and frequency modulation are shown in figure 7*a–e*. Within the low-pitch calls, three clusters can be further identified (cluster 0, 4 and 5). Calls belonging to cluster 5 are the rarest at all ages and are characterized by extremely low-frequency modulation (6.2 kHz ± 1.7), short duration (0.034 s ± 0.005) and low amplitudes (32.48 dBFS ± 2.52), illustrating the emission of low-pitched, soft, short and simple sounds (figure 7*a–e* and interactive electronic supplementary material). The other two subclusters (0 and 4) within the lower pitch group show the typical medium-to-high frequency modulation (52.7 kHz ± 4.7 and 50.2 kHz ± 2.6, respectively), but vary in duration and amplitude, both showing relatively high values (duration: 0.085 s ± 0.005 and 0.068 s ± 0.004; amplitude: 62.75 dBFS ± 2.93 and 45.47 dBFS ± 2.42). These clusters illustrate the existence of more complex, lower-pitched sounds. The higher frequency band can be subdivided into two main clusters (2 and 3) that show similar peak frequencies at all ages (figure 7*b* and interactive electronic supplementary material). Cluster 3 is the loudest within the high-pitch call group (43.80 dBFS ± 4.42) with medium duration (0.060 s ± 0.003) but relatively low-frequency modulation (18.5 kHz ± 2.4). A minority of calls within this cluster extend to lower frequency ranges. Instead, calls of cluster 2 are soft, simple calls with a low amplitude (39.85 dBFS ± 2.63), extremely short call durations (0.025 s ± 0.003) and very low-frequency modulation values (8.2 kHz ± 1.5). One cluster (cluster 1) spans the entire frequency range and is characterized by lower amplitudes (32.48 dBFS ± 3.86), medium duration (0.066 s ± 0.005) but extremely high-frequency modulation (66.6 kHz ± 5.0), hinting at the emission of relatively quiet but intense and complex sounds. Throughout development, all clusters remain present although the relative contribution of each cluster differs throughout development (age × cluster *p* < 0.0001, figure 7*f*; electronic supplementary material, figure S6). Moreover, peak frequency, call amplitude and frequency modulation change in a cluster-dependent manner throughout development (cluster × age *p* value peak frequency < 0.0001, duration *p* = 0.011, amplitude *p* < 0.0001, frequency modulation *p* < 0.0001). As higher-pitched calls become more prevalent over time, the extension of clusters 3 and 1 into lower frequency ranges diminishes (see interactive electronic supplementary material). This increase of high-pitched calls can be mainly attributed to an increase of calls belonging to cluster types 2 and 3 and a decrease of calls belonging to clusters 0 and 1.

When comparing the distribution of the call clusters between genotypes over development, some slight overall differences can be observed (figure 7*g* and *p*-value cluster 3 = 0.0257 (Wt–Ho)). Nonetheless, no genotype effects were significant when analysing the ages individually (electronic supplementary material, figure S7). To investigate whether loss of *Pcdh10* affected socio-affective communication with the mother, maternal retrieval time was analysed in our conditional KO model. In this model, the mother is always wild-type. As some pups tended to independently crawl back to their nest, especially at later developmental stages, retrieval latency was only taken into account if they were retrieved by their mother. Developmental age and genotype directly affected the retrieval time, with an additional shift in the retrieval time developmental trajectory by genotype (*p* age > 0.0001, *p* genotype = 0.013, *p* age × genotype = 0.0002; electronic supplementary material, figure S8).

## 4. Discussion

Here, we investigated the expression and function of *Pcdh10* in the developing rodent brain*,* a cell adhesion molecule linked to the development of ASD in humans [87]. We used a combination of interneuron-specific conditional and ubiquitous *Pcdh10* KO mice to zoom-in on certain neuronal cell populations, anxiety and socio-affective communication. In the conditional *Pcdh10* KO model, we observed reduced interneurons in the BLC of the amygdala and altered socio-affective communication, implicating interneurons in ASD aetiology.

### (a) Throughout development Pcdh10 is expressed in brain regions linked to autism spectrum disorder and social behaviour

Involvement of members of the non-clustered protocadherin family in proliferation, sorting and spatial patterning of the brain has become evident over recent years [88–91]. Non-clustered protocadherins are associated with neural circuit formation [92], and *Pcdh10* has been directly implicated in the wiring of olfactory and amygdalar circuitry [34,36,93,94]. The combinatorial expression of cadherin superfamily members, including protocadherins, delineates amygdala subdivisions, and this was suggested to underlie the functional organization of the amygdala [95]. We found *Pcdh10* to be preferably expressed in developing structures of the basal ganglia, olfactory and limbic systems. Brain regions that show strong *Pcdh10* expression overlap significantly with regions functionally linked to emotional regulation, fear and social behaviour [96–102]. Perturbed expression of *Pcdh10* could therefore result in circuit-level dysfunctions manifesting as ASD-associated behaviour.

The presence of *Pcdh10* in these brain structures already at early development stages suggests its role is not only confined to the maintenance of neural circuits but also extends to the early establishment of these structures. PCDH10 levels are already high at E13.5 and continue to rise during embryonic and early postnatal development, pointing towards an increasingly important role during neurogenesis, neuronal migration, axonal outgrowth and synapse formation and elimination. Expression of *Pcdh10* within synapses of the amygdala at juvenile and adult stages was previously reported [34,36], and *Pcdh10* was linked to synapse elimination in hippocampal cell cultures [103]. Our data show that *Pcdh10* is already strongly expressed in the BLC of the amygdala during embryonic development (E17.5), but that the preferential expression within the BLA becomes stronger after birth. This dynamic change could imply that *Pcdh10* is involved in the early development of the entire BLC, but is then restricted to the BLA to regulate circuit formation and maintenance. Abnormal synapse formation or maintenance has been implicated in ASD pathophysiology [87,104]. Synaptic dysfunction and improper excitatory/inhibitory balance both affect neuronal transmission, suggesting that disrupted communication between key neuronal groups is fundamental to ASD development. At juvenile stages, both excitatory and inhibitory synapses in the BLC express *Pcdh10* [34].

### (b) PCDH10 is linked to the synaptic department and proteasome

To explore the molecular function of PCDH10 at the (synaptic) membrane, we created a novel *Pcdh10*-HAV5 mouse line that allowed for mass spectrometry identification of endogenous PCDH10-binding partners. Here, multiple proteins were identified that could be linked to the pre- and post-synapse, in addition to proteins linked to actin dynamics and ubiquitination. The analysis of the scRNA dataset of the adult brain likewise revealed a co-occurrence with genes involved in synaptic organization, maintenance and plasticity. A previous study has indicated a role for *Pcdh10* during activity-dependent synapse elimination at the age that we performed Co-IP (P6–P7) [103]. At P14 and P28, *Pcdh10* KO mice show a reduced density and size of excitatory pre- and post-synapses in the BLA [34]. Moreover, *Pcdh10* remains strongly expressed in both excitatory and inhibitory pre- and post-synapses at P28, at the end of synaptic development in the rodent, and in adulthood [34,36]. Our analysis of adult scRNA datasets yields additional arguments that *Pcdh10* remains involved in the synapse at adult stages. Intriguingly, in the first experiment using V5 beads, we identified NMDA receptor subunit NR1. A previous study has shown that adult mice haploinsufficient for *Pcdh10* contained a decreased amount of GluN1 and GluN2 in their amygdala, two subunits of the NMDA receptor [36]. The abnormal social behaviour of males could be rescued with the injection of the NMDAR glycine site partial agonist d-cycloserine (dCS) [36,49]. This indicates that PCDH10 somehow regulates the expression, translation or the stability of these NMDA-R subunits in the amygdala. In addition to synaptic proteins, we identified proteins involved in ubiquitination and proteasomal degradation (PSMD1, MycBP2). PCDH10 contains a proteasome interacting region within its cytoplasmic domain and *Pcdh10* expression is required to link ubiquitinylated PSD-95 to the proteasome during synapse elimination [103]. Within the amygdala proteolytic activity was found to be paramount for fear conditioning processes and the formation and stability of long-term fear memory, and here proteasome activity was NMDAR-dependent and upregulated downstream from CAMKII signalling [105–108]. Taken together, PCDH10 seems to bind proteins involved in synaptic transmission and plasticity, and proteins involved in proteolytic homeostasis and mechanistic target of rapamycin (mTOR) signalling. Further validation of these binding partners could yield insight into the molecular mechanism of PCDH10 deficiency.

### (c) Reduced amount of interneurons in the basolateral complex of the Gsh2:cKO mouse model

Our study confirmed the expression of *Pcdh10* predominantly in LGE and CGE at embryonic stages. Strong expression was also observed in some structures located in the ventral telencephalon, including the BLC of the amygdala. The amygdala consists of several nuclei that could be considered predominantly ‘pallial’ or ‘subpallial’ in origin [109]. Previous studies have focused on the role of *Pcdh10* in excitatory neurons of the BLC during postnatal stages. However, our results indicate that already at embryonic stages, both excitatory neurons and G*sh2*-lineage interneurons express *Pcdh10* in the BLC.

Loss of *Pcdh10* within this subpopulation of interneurons caused a significant reduction in their numbers in the BLC of the amygdala at E17.5. In this conditional KO model, the excitatory cells of the BLC remain positive for *Pcdh10* while *Gsh2*-lineage interneurons lose this expression. This targeted loss specifically in interneurons might have compromised integration into the *Pcdh10-*positive BLC. Previous studies have revealed a strong association between several non-clustered protocadherins and sorting defects observed both *in vitro* and in the developing brain, including the formation of specific aggregates owing to differential δ2-protocadherin expression *in vitro* [90]*,* sorting anomalies and reduced interneuron migration in the cortex of *Pcdh19* mosaic females [90,92] and the mislocalization of *Gad1-*expressing cells in the thalamus of *Pcdh10b* knockdown zebrafish [110]. These findings collectively suggest that a quantitative mismatch of protocadherin expression in adjacent cells might affect their positioning and circuit integration. In the basolateral amygdala, such a mismatch might induce disturbance in the cell arrangement and circuit integration associated with aberrant USV emission. Alternatively, a proliferative or apoptosis defect in the progenitor/mature interneuron population could also explain the reduced interneuron numbers. In cancer, *PCDH10* has been linked to both these processes [25], but in the telencephalon, we could not observe an overall reduction of *Gsh2-*lineage interneurons at E17.5.

### (d) Ultrasonic vocalizations

A reduced number of interneurons could potentially disturb the balance between excitation and inhibition and impair BLC functioning, which we measured using USV as a readout. Isolation-induced USV are sometimes compared to the crying of human babies, as both serve a similar purpose [111]. In infant individuals diagnosed with ASD and in mouse models, abnormal crying and isolation-induced USV have been observed [11,12,111,112]. While reduced pup ultrasonic calling is typically interpreted as evidence for socio-affective communication impairment [8,11,12], increased emission rates are often interpreted as an indirect measure of enhanced anxiety levels [85,86].

An inverted U-shaped developmental call emission pattern could be identified for both the KO and the cKO, as previously described in mice [16–18]. Hoshina *et al*. [34] have reported decreased USV emission in homozygous *Pcdh10* KO as compared to wild-type pups [34]. They postulated that the increased USV previously observed by Schoch *et al*. [36] in the heterozygous KO mice might be caused by non-specific effects of the LacZ cassette [34,36]. However, in our study, both ubiquitous as well as conditional heterozygous models also exhibited heightened USV emission rates, most prominent during the peak call emission period (P3–P6). In contrast, homozygous Gsh2:cKO pups showed decreased instead of increased pup ultrasonic callings at P12, revealing a dynamic and more complex involvement of *Pcdh10*. Future studies should investigate whether ubiquitous heterozygous and homozygous *Pcdh10* KO pups show varying USV phenotypes. A majority of the genes linked to ASD are described as gene-dosage sensitive genes, and point mutations and/or copy number variations (including both deletions and duplications) affecting only one allele are common in individuals with ASD [87]. For some genes, both individuals with deletions and those with duplications have been described to suffer from ASD, although the specific symptoms can be distinct [87] and transcriptomic changes related to ASD might appear at different life stages [113]. These findings highlight that the function of ASD-related genes might be dynamic throughout life and might be gene-dosage sensitive.

Previous studies on isolation-induced USV in mice and rats showed that calls can be divided into distinct clusters of call subtypes based on their peak frequency, call duration, amplitude and frequency modulation [11,17,114–116]. Mothers were observed to show categorical frequency perception for certain frequency bands, indicating that emission of calls with abnormal peak frequency might affect care and retrieval behaviour [13]. We identified six call subtypes (clusters), defined by peak frequency, duration, amplitude and frequency modulation, which could provide additional insight into the infant mouse communicative potential.

Calls cluster in two peak frequency ranges, including low-pitch calls around 60 kHz (55–65 kHz) and high-pitch calls around 80 kHz (70–100 kHz). Low-pitch calls were predominantly longer, more complex and louder as compared to high-pitch calls. In previous experiments in mice and rats, the high-pitch call type was described to have low-frequency modulation and call duration, similar to our call clusters 2 and 3, while the two low-pitch call types could be divided into one with low-frequency modulation and one with high-frequency modulation, similar to our cluster 0 and 4 versus 5 [115,117]. We have further subdivided these call types and identified an additional group with high pitch and high-frequency modulation (cluster 1) in CD-1 mice. The prevalence of the high-pitch calls increased over development, as also previously observed [16,17,115,116,118]. These two peak frequency clusters are also clearly present in pups heterozygous for *Pcdh10* KO and *Pcdh10* cKO, although the peak frequencies are subtly shifted towards lower values with increased frequency modulation in pups heterozygous for conditional KO of *Pcdh10* in *Gsh2*-lineage interneurons. Clusters become more comparable to wild-type patterns as development progresses. The behavioural implications for these changes remain largely unknown, but genotype affected the retrieval behaviour of the mother. Since the contribution of the more complex isolation-induced calls decreases over development, they might primarily serve to induce a rigid retrieval response in the mother as compared to calls with lower frequency modulation.

In mammals, multiple brain regions are involved in vocal communication, including the anterior cingulate cortex, the periaqueductal grey (PAG), the reticular formation, the motor cortex, basal ganglia, cerebellum and phonatory motoneurons [119]. In the conditional *Pcdh10* KO model, we observed reduced interneurons in the BLC of the amygdala. Changes in the socio-affective communication are more pronounced in the conditional KO model as compared to the ubiquitous knockout, implicating the targeted interneuron subpopulation in this phenotype. Abnormal interneuron function and decreased gamma-aminobutyric acid (GABA) transmission in the amygdala directly affects sociability and fear behaviour in rodents [47,120,121]. In addition, USV have been linked to both anxiety and the amygdala [85,86,122–124]. In mice, the amygdala was shown to affect USV behaviour in the context of courtship via connections to the PAG, which weighs the USV-promoting signals from the POA against the USV-suppressing, GABAergic signals emerging from the amygdala [125]. Reduction of GABAergic input from the amygdala, for example, owing to reduced interneurons as observed in our model, could, therefore, directly affect the gating of vocalization behaviour in the PAG.

Analysis of the adult brain scRNAseq dataset revealed that *Pcdh10*-expressing cells differentially expressed genes involved in vocalization behaviours, including *Foxp2, Cntnap2, Nrxn1* and *Nrxn3,* as compared to cells that did not express *Pcdh10.* This might indicate that *Pcdh10* generally functions in brain regions linked to vocalization. *Gsh2* is also strongly expressed in the hindbrain at early developmental stages [126], potentially targeting some of the known vocalization nuclei. Investigation of these regions should reveal whether the observed phenotype primarily is the result of reduced *Pcdh10* in the interneurons of the basal ganglia and amygdala, or if other (hindbrain) regions are involved.

### (e) Limitations of the study

Studies on USV in *Pcdh10* KO lines have previously been performed, and similar results were obtained in heterozygous pups of the *Pcdh10* ^LacZ/+^ line used by Schoch *et al*. [36]. Although the overall statistical significance and difference between the number of calls in heterozygous KO pups and wild-types is rather modest (at P6 *p* = 0.041), these findings replicated previously published data and can, therefore, be considered robust. We did not test ubiquitous homozygous *Pcdh10* KO pups, complicating comparison with the recent publication by Hoshina *et al*. [34]. The effects on USV numbers were similar between our tested models, with the heterozygous cKO pups showing an exaggerated effect as compared to the ubiquitous heterozygous KO pups. This is demonstrated by the very low *p*-values for the age × genotype interaction term (*p* < 0.0001) and modest *p*-values for the wild-type versus heterozygous comparison at P6 (*p* = 0.010). Combined with the robust (replicable) effect seen in the ubiquitous knockout, we can conclude that the conditional KO shows a similar or stronger effect on USV emission.

The analysis of distinct clusters of call subtypes revealed similar findings as previously published; however, we further divided calls into six different subtypes. We want to highlight that this spectral clustering of call types is likely still an underrepresentation of the vocal repertoire of isolated pups, and that some clusters can be further divided into subclusters. The clusters identified in this publication can be used as a general classification of call types based on selected parameters, and serve as a guide for studies that want to further characterize the communicative potential of isolated neonatal pups.

While we show a robust effect of the conditional loss of *Pcdh10* on the amount of interneurons in the amygdala (control: 24.27% ± 2.44, cKO: 10.12% ± 4.61, *p* = 0.002), we do not claim or prove that this specific reduction in the amygdala is the major cause of the altered isolation-induced USV behaviour in the conditional KO model. As mentioned in the discussion, *Pcdh10* is co-expressed with other genes involved in vocalization and might be strongly expressed in *Gsh2-*lineage interneurons of other vocalization nuclei. Nonetheless, the specificity of the conditional KO model proves that inhibitory interneurons are involved in this phenotype, and highlights that *Pcdh10* expression can regulate the ratio of excitatory and inhibitory neurons in at least the BLC. An altered excitation/inhibition balance in the amygdala has previously been linked to altered sociability and anxiety in rodents.

## 5. Conclusions

This study highlights the involvement of *Pcdh10* in anxiety-related behaviour and socio-affective communication in developing mice pups and implicates interneuron subpopulations in the behavioural alterations relevant to ASD. *Pcdh10* is strongly expressed in interneurons and expression increases over embryonic and early postnatal development. Targeted deletion of *Pcdh10* in *Gsh2*-lineage interneurons revealed a reduced presence of this subpopulation in the BLC of the amygdala as compared to controls. Isolation-induced USV were altered in heterozygous pups, which exhibited increased emission rates and altered acoustic features including decreased peak frequencies and enhanced frequency modulation. This phenotype is similar to and even stronger than that of a ubiquitous heterozygous knock-out of *Pcdh10.* Together with the identification of distinct clusters of call subtypes and their subtle variations between genotypes, this emphasizes the complex role of *Pcdh10* in vocal communication and anxiety, two aspects of behaviour often affected in ASD.

## Data Availability

All data are available in this published article, in the electronic supplementary material [127] and in the GitHub repository [128].
